# High Performance Polymer Composites: A Role of Transfer Films in Ensuring Tribological Properties—A Review †

**DOI:** 10.3390/polym14050975

**Published:** 2022-02-28

**Authors:** Sergey V. Panin, Vladislav O. Alexenko, Dmitry G. Buslovich

**Affiliations:** 1 Laboratory of Mechanics of Polymer Composite Materials, Institute of Strength Physics and Materials Science of Siberian Branch of Russian Academy of Sciences, 634055 Tomsk, Russia; vl.aleksenko@mail.ru (V.O.A.); buslovich@ispms.ru (D.G.B.); 2 Department of Materials Science, Engineering School of Advanced Manufacturing Technologies, National Research Tomsk Polytechnic University, 634050 Tomsk, Russia

**Keywords:** high-performance polymers, antifriction composites, transfer film, wear rate, coefficient of friction, solid lubricants, fibers, nanoparticles, adhesion

## Abstract

The purpose of this review is to summarize data on the structure, mechanical and tribological properties, and wear patterns of composites based on high-performance polymers (HPPs) intended for use in friction units. The review includes three key sections, divided according to the tribological contact schemes regardless of the polymer matrix. In the second part, the analysis of composites is carried out in point contacts. The third section is devoted to the results of studies of HPP-based composites in linear ones. The fourth section summarizes information on flat contacts. Particular attention is paid to the formation of transfer films (TFs) in the contacts and their influence on the tribological patterns of the studied rubbing materials. As a conclusion, it is noted that the challenge of experimental methods for analyzing TFs, stated by K. Friedrich, is effectively solved in recent studies by the XPS method, which enables us to accurately determine their composition. Although this determination is completed after the tribological tests, it allows not only a more accurate interpretation of their results considering specific conditions and loading schemes, but also the ability to design HPP-based composites that form required TFs performing their preset functions.

## 1. Introduction

The purpose of this review is to summarize data on the structure, mechanical and tribological properties, and wear patterns of composites based on high-performance polymers (HPP) intended for use in friction units. A number of such materials are described in excessive detail in the literature, in particular, neat polyetheretherketone (PEEK) and PEEK-based composites, while a similar polyaryletherketone (PAEK) polymer has rarely been the subject of research. At the same time, polytetrafluoroethylene (PTFE) is not included in this list as a matrix material, despite the fact that it is extremely widely used as solid lubricant filler. The motivation for this critical review has been the highly ambiguous published data on the effect of the same types of fillers on the tribological properties of composites, which is illustrated many times below. The authors are aware that such an effect is associated primarily with the difference in conditions of tribological tests, including a loading scheme (a contact type), load-speed (P·V) parameters, temperatures and environments, and the counterpart material and its roughness, etc. For this reason, the conditions of the tribological tests are characterized in the description and analysis of the obtained results.

As a key criterion (the top level of the classification), a contact scheme of the polymer composites and counterparts (namely, point, line, and flat ones) is adopted since it determines the macroscale level of the tribological interaction of the rubbing bodies. The influence of other parameters is considered when interpreting reported values of the coefficient of friction (CoF) and wear rate (WR), as well as the characteristic mechanisms that determine an increase/decrease in wear resistance.

The prospect of using HPPs to design materials for friction units is determined by their two key properties: high melting points and great strength properties. In addition to an acceptable level of melt flow index and the ability to provide interfacial adhesion to filler particles/fibers (including at a sufficiently high degree of their content), these materials are also attractive because of their ability to process and improve the stiffness/load-bearing capacity. However, advanced strength properties of this class of materials simultaneously determine great CoF levels, which in most cases can exceed 0.3 on steel under dry sliding friction (DSF) conditions. According to Brian J. Briscoe, a typical method for solving this problem is loading polymers with various types of fillers using the ‘hard and strong fillers in a softer matrix’ or ‘soft and lubricating fillers in a hard and strong matrix’ principles [1].

Recently, special attention has been paid to loading metals and nonmetal oxides (in particular, SiO_2_, SiC, ZnO, TiO_2_, Al_2_O_3_, Si_3_N_4_, CuO) with nanoparticles as a way to improve the load-bearing capacity and wear resistance of the polymer composites against counterparts [2]. In addition, a large number of researchers studying the composites have loaded carbon nanotubes (CNTs) with the goal of enforcing polymers since CNTs show very high strength and stiffness.

Regardless of the types of loaded fillers, the invariable condition for enhancing wear resistance is the formation of a thin and uniform transfer film (TF) or transfer film layer (TFL) and its reliable adherence on a counterpart. Thinner TFs tend to adhere stronger to counterpart surfaces than thicker ones. In this regard, the emphasis in interpreting the results of tribological tests of polymer composites is shifting toward the specifics of the TF formation and fixation on the counterparts. Additionally, there are two conditions that determine the ability of a polymer composite to form such a film: (1) loss of the polymer material by attrition due to interaction with metal asperities; and (2) its ability to be adhered and retained on the counterpart surface for a long time. The latter process is hindered by the loss of the TFs via debonding from the counterparts [2]. A third important effect on the wear process should also be added, namely, the formation of oxidized debris in the tribological contact zones, including from the fractured TFs. They can both exert an abrasive effect and act as a third body that facilitates sliding.

**Figure 1:**
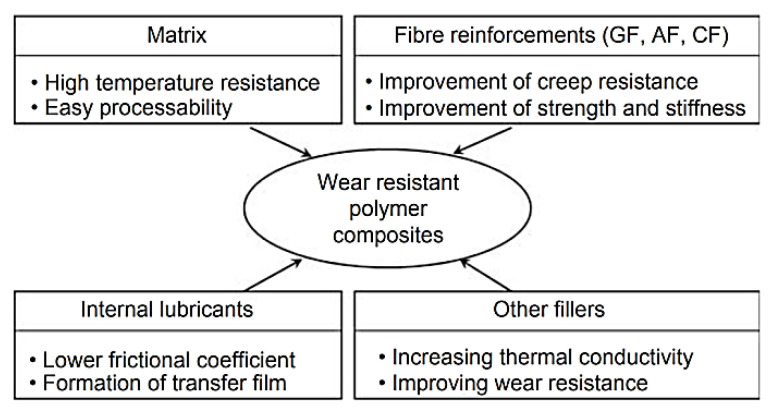
A summary of the structural components of the wear resistant polymer composites. Figure is reproduced with permission from reference [3].

As an effective approach for designing antifriction polymer composites, Li Chang [3] analyzed the formation of hybrid materials that simultaneously include both solid lubricant particles and reinforcing fibers. The synergetic principle of their action (used in the interpretation of the data of at least 15% of publications on the subject of this review) characterizes a scheme in Figure 1. The following are noted as key tribological aspects [3]: (1) The pin-on-disc (P-o-D) and block-on-ring (B-o-R) schemes are two generally used methods for sliding wear tests. (2) The presence of reinforcing fibers strengthens the polymer composites and protects them against irregularities (asperities) on the counterpart surfaces on the one hand. On the other hand, hard fibers can damage the counterpart surfaces and prevent TFs from attaching to them. Finally, the author concluded that ‘understanding of the growth of TFLs and their tribological behavior during steady state wearing stage is still limited owing to the lack of quantitative techniques’ [3]. Note that researchers of HPP-based composites put very different concepts into the TF term despite the obviousness of the wear process development. For this reason, the TF role in the formation of the tribological properties can be both negative and positive, even providing friction in the ‘wearless’ mode.

In [4] Meghashree Padhan suggested some examples of hybrid ‘PEEK + PAEK’-based composites loaded with 30% short carbon fibers (SCFs) and 10% graphite (Gr) nanoparticles as primary solid lubricants (SLs), as well as 10% noncarbon secondary SLs such as WS_2_, MoS_2_, h-BN, etc. When interpreting the results, the effect of synergism between both types of SLs and the best efficiency of nanoparticles over microsized inclusions were considered.

The widespread use of nanofillers to improve wear resistance of polymer composites has prompted a significant number of reviews. For example, Qihua Wang emphasized [5] that nanoparticles alter friction and wear behavior in different ways. As a result, low CoF levels do not necessarily correspond to decreased WR values. At the same time, the factors affecting wear resistance are types of the combined polymer and nanoparticles, the filler content, and the friction contact size and shape in addition to operating conditions. Due to so many influencing factors, the effect of nanoparticles on the tribological properties of the antifriction polymer composites is still an open field for further research. This is especially the case when nanoparticles in combination with conventional fillers are considered. In doing so, a detailed analysis of the tribological behavior of various types of the composites with explicit consideration of the specific conditions of tribological tests is required.

**Figure 2:**
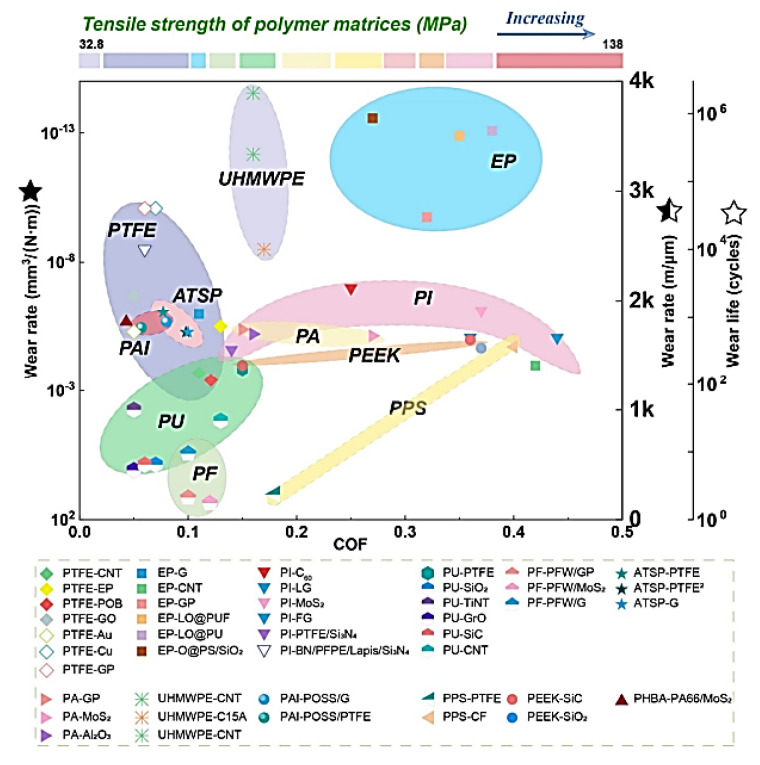
The tribological properties of different polymer matrices and fillers. Figure is reproduced with permission from reference [7].

A modification of M. Ashby’s concept on ranking structural materials over multidirectional functional properties [6] has been suggested for designing polymer composites for tribological purposes [7]. It considers WR, CoF and tensile strength levels of the polymer matrix, etc. (Figure 2). This approach is important due to its versatility since it enables the solution of the practically important issues of choosing polymer composites (including multicomponent ones) for given operating conditions. However, as noted above, the tribological properties depend on a number of parameters, so it is almost impossible to offer a completely universal approach within the framework of such data. As a result, a promising research area is the implementation of machine learning algorithms [3].

The demand for HPPs in various industries has prompted a variety of reviews on their functional properties. In particular, Kurdi, A. et al. discussed the wear mechanisms under sliding friction for designing high-performance composites [8]. In this case, special attention was also paid to environmental factors, namely lubrication conditions, temperature, and even the possibility of developing vibration-induced phenomena. It was noted that loading with fillers may cause discontinuities in the polymer matrix, which promote the formation of debris. As a result, the critical filler contents should be limited to 5–15 vol.%. The role of nanoparticles in changing wear resistance narrows to both probable rolling effect and topographic smoothening of the friction surfaces. Similar to other authors in this field of science, Kurdi, A. and Chang, L. noted that the desired effect is achieved mainly by using a combination of reinforcing particles and fibers. However, an increase in both strength and toughness of HPPs does not always reduce the intensity of the wear mechanism development and the formation of debris.

**Figure 3:**
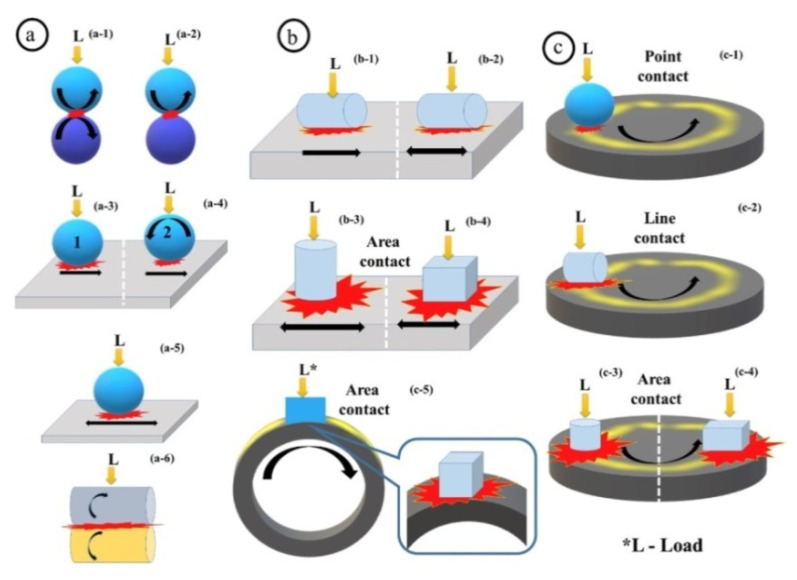
Various tribo-configurations and tribo-contacts: point contact (**a-1** to **a-5** and **c-1**), line contacts (**a-6**, **b-1** and **c-2**) and area contacts (**b-2**, **c-3** and **c-4**). Rotary motion (**a-1**, **a-2**, **a-4**, **a-6**, **c-1**, **c-2**, **c-3**, **c-4** and **c-5**), Reciprocating linear motion (**a-5**, **b-2**, **b-3**, **b-4**), linear forward motion (**a-3**, **a-4** and **b-1**). Figure is reproduced with permission from reference [4].

Neat HPPs are not antifriction materials due to their high strength, which inhibits easy sliding on the counterpart (primarily steel) surfaces., This challenge is overcome in the design of composites by loading solid lubricant particles, followed by TF formation on the counterparts. However, the number of asperities on the sliding surfaces, determining CoF levels, also depends on the contact area. Three contact types are typically distinguished in tribology: point, line, and area (Figure 3) [4]. It is not too difficult to form a TF that is uniform in thickness and relatively firmly adhered on the surface of a steel ball at a point contact; however, this task becomes highly complicated for area contact types. The reason is lower specific pressures for this tribological loading scheme (as a rule). Temperature is another important aspect, since it is easier to realize heating followed by tribological oxidation and adhesion in a point contact than in both line and area ones. Due to this fact, the tribological test schemes have been accepted as the classification principle in this review.

HPPs include polyethersulfone (PES), polyetherimide (PEI), polyphenylene sulfide (PPS), PEEK, fluoropolymers, etc. Their attractiveness lies in the possibility of replacing metal parts with nonmetallic ones in high-tech industries [9]. However, most papers on HPPs for tribological applications focus on a limited number of thermoplastics such as PPS, PEEK, and polyimide (PI). In this regard, numerous loaded fillers, their combinations, as well as schemes and conditions of tribological tests should be investigated on this topic.

Guowei Chen named CFs, CNTs, Gr, graphene (GN) and carbon black as the key fillers for PPS in his review [10]. Aspects of using biocarbon as an alternative for petroleum-based CFs loaded in PPS-based composites were also discussed. Some patterns of designing, processing, and studying functional properties of the PPS-based composites were also summarized in the review [11].

A significant number of papers on the use of PEEK-based composites are devoted to orthopedic applications. The authors are looking for ways to replace metal implants (components) with polymer ones, for which neat PEEK is often loaded with CFs [12]. In the design of PEEK-based composites for biomedical engineering applications, aspects of the polymer-filler interfacial interaction and their fabrication methods also play an important role. Oladapo et al. [13] noted perspectives of the PEEK-based composites from the point of view of the implementation of additive manufacturing (AM) procedures. Also, cellular calcium hydroxyapatite (CHAp) was analyzed as an advanced excipient. Another review [14] is devoted to the use of PEEK for designing biomedical composites and highlights its relevance in dental applications as well. In addition, PEEK is widely used for manufacturing knee, hip, spine and other implants in orthopedics. A review on dental applications of PEEK is given in [15]. Further analysis of the issue in its application for scaffolds was carried out by the authors of [16]. In a similar paper [17] Ma, H. et al. summarized some aspects of the performance requirements, the composite design process, and the surface modification when using PEEK as a material for the manufacture of orthopedic implants.

**Figure 4:**
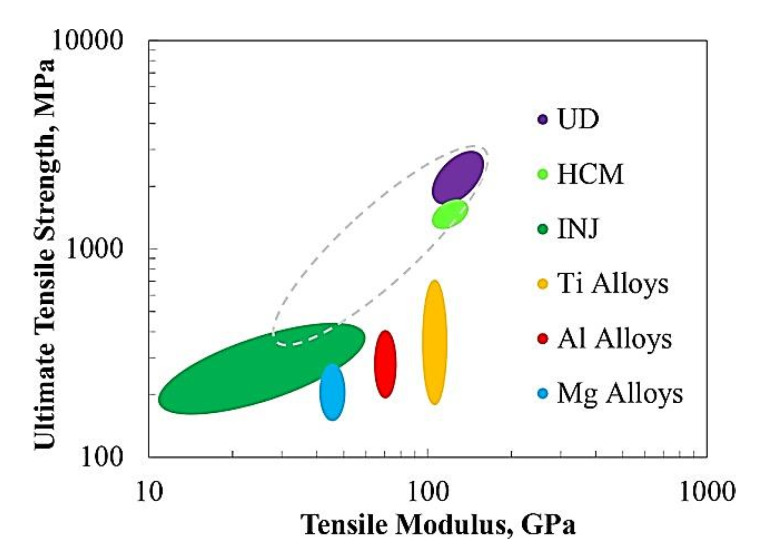
Typical tensile properties of common metallic alloys and CF-reinforced PAEK-based composites: UD, unidirectional fibers; HCM, hot compression molded fibers; INJ, injection molded fibers. Figure is reproduced with permission from reference [19].

Because the tribological properties of the polymer composites are determined by their friction surfaces, some data on the PEEK surface modification have also been reviewed. In [18], Singh, S. analyzed plasma treatment of PEEK in terms of its influence on the biological, surface (adhesion and wettability), mechanical, and tribological properties. As noted above, some similar materials are gaining popularity in the development of HPP-based composites. In their review [19] Veazey, D., et al. proposed a roadmap for high-performance PAEK-based composites reinforced with long fibers (Figure 4). They emphasized that one of the key challenges in designing these composites is improving the fiber-matrix interfacial bond strength.

The unique properties of PI have determined the prospects for some practical applications of PI-based composites. The most detailed review [20] covers the issues of their synthesis. It has been shown that the presence of functional groups and the formation of dendritic structures enable unique properties in PI-based composites. Their key advantage is the possibility of high-temperature applications. Nevertheless, some design challenges remain for the PI-based nanocomposites. For example, Ogbonna, V.E. emphasized [21] some issues of interfacial adhesion and surface degradation, which affect their mechanical properties and abrasive wear resistance in tribological applications. The following are considered fillers for such PI-based nanocomposites: CNTs, GN, graphene oxide (GO), boron nitride (BN), MoS_2_, silica (SiO_2_), titania (TiO_2_), alumina (Al_2_O_3_), CFs, aramid fibers (AFs), GF, zinc dioxide (ZnO_2_), zirconium dioxide (ZrO_2_), silicon nitride (Si_2_N_4_), and carbon nitride (C_3_N_4_). In [22] nanofillers of the mentioned types were reviewed from the standpoint of their influence on the structure and mechanical properties of the PI-based nanocomposites. Also, similar to the PEEK-based composites, the PI-based ones are used in medical applications. Some chemical, physical, and manufacturing aspects of designing the PI-based biocompatible composites are summarized by Catalin P. Constantin in [23].

This review consists of three key sections, divided according to the tribological contact schemes regardless of the polymer matrix. To facilitate comparison and interpretation of the presented data for each cited paper, the terms of the tribological tests are briefly outlined. This slightly complicates the possibility of generalizing the data but enables us to better identify the specific features of the obtained results.

## 2. Point Contacts

### 2.1. Neat HPP

**Figure 5:**
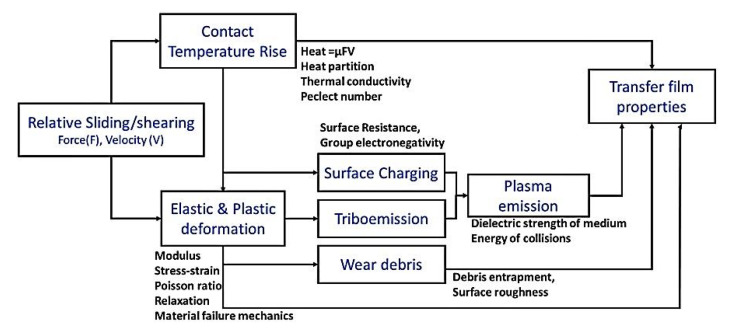
Factors effecting functional properties of polymeric TFs upon sliding PEEK on the steel surfaces. Figure is reproduced with permission from reference [24].

In [24] Puhan, D. et al. tested neat PEEK on both steel and sapphire counterparts. It showed that the chemical compositions of the formed TFs differ from that of neat PEEK. The TFs mainly consisted of amorphous carbonaceous materials. Molecular chains of PEEK were broken in different positions of the ester and ketone groups. Additionally, opening of aromatic rings, substitution, and cross-linking, as well as both crystallinity and coplanarity losses were reported. As shown in Figure 5, a carboxylic acid formed, the interaction of which caused the formation of thin and strong TFs during friction with the surfaces of the steel or sapphire counterpart *(PEEK; R_a_* = *1–1.2 µm; B-o-D; counterpart is a ball with a diameter of 6 mm; R_a_ = 0.02 µm; P = 10 N; V = 2 m/s; L = 3.6 km; P_max_ = 160 MPa).*

A number of papers are devoted to the effect of various surface treatment procedures on the tribological properties of PEEK. For example, Wang, M. showed the influence of engineered surface microstructuring on friction patterns in PEEK-steel contacts [25]. Pronounced fluctuations of CoF curves were observed in both nontextured and textured PEEK containing microholes with a diameter of 25 μm. This was accompanied by the formation of a noticeable amount of debris. In the case of friction of the GCr15 steel ball on the microtextured PEEK surface with holes 50 μm in diameter, the WR level was reduced sharply *(PEEK; GCr15 ball; RF; R_a_ = 0.02 µm; B-o-F; DSF; LF = 0.9 and 3 N).*

Cho, M. investigated the ability of PPS to form a TF while sliding on steel [26]. The steel counterpart surfaces were textured with four pore densities of 5, 15, 25, and 35% using a laser. Then, pores were filled with PPS powder followed by curing. This hybrid texturing protected the steel surfaces with formed TFs. An effective decrease in CoF levels was observed for the surface with the pore density of 5% at V = 0.15 m/s, in contrast to both V values of 0.05 and 0.10 m/s since high sliding speeds favored TF formation. In the case of the pore density of 15%, the CoF values were decreased for all sliding speeds, because TFs of moderate thicknesses covered all wear tracks *(PPS; B-o-D; 100Cr6 steel ball; R_a_ = n/d; DSF; V = 0.05, 0.10, and 0.15 m/s; P = 9.8 N; L = 360 m)*.

Duan, C., et al. analyzed aspects of the influence of the PI molecular structure on the mechanical and tribological properties under conditions of incorporation of 4,4′-ODA into polyimide with 3,4′-ODA isomer [27]. It was shown that CoF levels were lowered with raising the 4,4′-oxydianiline (4,4′-ODA) content in the PI macromolecular chains. These changes were related to the macromolecular chain conformation caused by different monomer configurations. Thus, PI with paraposition diamine exhibited the best tribological properties *(PI; B-o-F; RF; counterpart is the GCr15 steel ball with a diameter of 3 mm; R_a_ = n/d; P = 5 N; V = 0.1 m/s; Ampl = 2.5 mm; T = 20 °C; RH = 16–20%; L = 300 m).*

In [28] Fareed, M. I. studied the tribological properties of PEEK by varying its structure through various heat treatment procedures such as annealing, normalizing, and both water and oil quenching from a temperature of 250 °C. The PEEK crystallinity reduced with an increase in its cooling rate. At the same time, the lowest crystallinity was found after water quenching. Conversely, the annealed samples showed the highest wear resistance because of the enhanced crystallinity. For each of these heat treatment procedures, WR levels increased with rising normal load *(PEEK; B-o-D; Stainless steel ball; R_a_ = n/d; P = 30, 60, 90 N; V = 62.8 mm/s; L = 62.8 m; DSF).*

**Figure 6:**
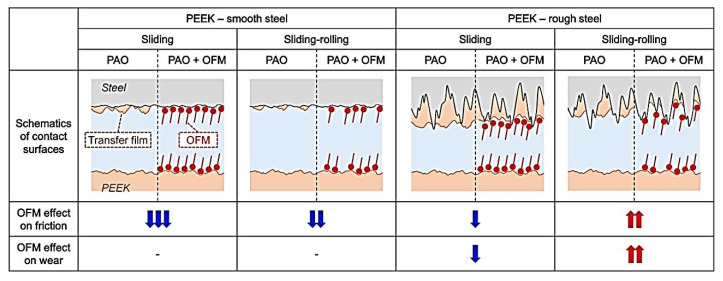
The OFM lubrication mechanism in the PEEK-steel contacts. Figure is reproduced with permission from reference [29].

Lubricating medium in a tribological contact changes conditions of TF formation. Tatsumi, G., et al. evaluated the role of organic friction modifiers (OFMs) in the lubrication improvement in PEEK-steel contacts [29]. Compared with OFM-A (oleylamine) and OFM-B (oleic acid), OFM-C (N-oleoyl sarcosine) showed a significant reduction in CoF levels in the ‘PEEK-smooth steel’ friction pair by 200% under sliding conditions and by 50% under the sliding-rolling ones. A similar CoF reduction was observed for OFM-C in both ‘PEEK-PEEK’ and ‘steel-steel’ friction pairs. A clear correlation between thicknesses of the PEEK TFs and the tribological properties of the ‘PEEK-steel’ friction pair was found. OFM-C exerted a great impact on these characteristics due to its ability to be strongly absorbed on both materials (Figure 6), inhibiting PEEK TF formation. This had both positive and negative effects on the tribological properties depending on the test conditions *(PEEK; B-o-P; sliding/sliding-rolling; R_a_ = n/d; V = 1 m/s; P = 50 N (PEEK-steel and PEEK-PEEK); P = 5 N (steel-steel); T = 25 °C).*

An important aspect that determines the development of the tribological processes is temperature in the friction contacts. Laux, K.A., et al. used infrared thermography to observe the full field temperature map of PEEK upon its sliding on both stainless steel and sapphire counterparts [30]. It was shown that PEEK debris attaches easily to the steel counterpart but not to the sapphire one. The PEEK transfer to the steel surface may be accompanied by an increase in the sliding interface temperature (up to T_g_). The PEEK TF was essentially oriented towards the steel counterpart surface. For this reason, TF formation was determined by the friction processes and the tribological contact temperature, which are not functionally dependent on the load-speed parameters *(PEEK; B-o-D; counterparts are the sapphire ball with a diameter of 19 mm (R_a_ = 1.5 µm) and the 52,100 steel disc 46 mm in diameter (R_a_ = 10 nm); T = 25 °C; P = 1–40 N; V = 100 mm/s).*

**Figure 7:**
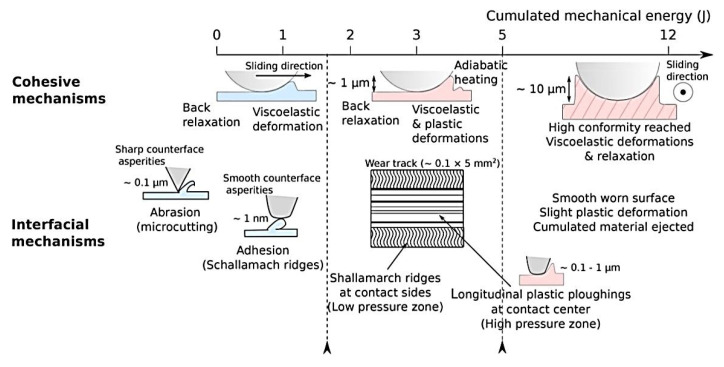
The cohesive and interfacial contact mechanisms as a function of the cumulated mechanical energy. Figure is reproduced with permission from reference [31].

Yahiaoui, M., et al. studied the tribological behavior of the PEEK-steel contact in reciprocating or unidirectional motion by varying load, speed, and sliding distance [31]. CoF values were reduced due to lowering the load that decreased the contribution of molecular attraction forces, as well as because of the rising speed that cumulated the mechanical energy. The reason was the adiabatic effect in the contact, which changed the polymer rheology. As a result, two interfacial mechanisms were identified (Figure 7). The first one was the formation of Schallamarch ridges due to nanoscopic interaction between them. The longitudinal ploughing mechanism was responsible for the formation of microscopic scratches. During reciprocal sliding, the polymer relaxation strongly affected its tribological behavior. Unidirectional sliding caused greater plastic strains and stretched Schallamarch ridges in the sliding direction *(PEEK; B-o-F; counterpart is the 100Cr6 steel ball with a diameter of 6 mm; reciprocating or unidirectional motion: (i) V = 100 mm/min, L = 250 mm, P = 1–30 N; (ii) P = 30 N, L = 250 mm, V = 10–500 mm/min; (iii) Ampl = 5 mm, L = 0.005–3.710 m; PEEK; R_a_ = 65 ± 9 nm).*

For HPPs, one of the limiting factors for wear resistance is the (T_g_) glass transition temperature. Jean-Fulcrand, et al. solved this issue by combining polybenzimidazole (PBI), having one of the highest T_g_ points, with PEEK in a ratio of 50:50 [32]. Chemical analysis of TFs formed on the steel counterpart surface and debris in the tribological contact showed the polymer degradation caused by shear heating. In the case of the ‘PEEK + PBI’ composite, TFs formed on the steel counterpart and possessed a composition similar to that of PEEK. When the contact temperature was close to the PEEK melting point, PEEK, as a composite component, formed a thin TF that acted as an interfacial lubricant. This reduced CoF levels, which in turn decreased the PBI degradation in the composite at high temperatures *(PEEK-PBI; B-o-D; T = 100, 190, and 280 °C; DSF; V = 0.1–2.0 m/s; L = 3.6 km; P = 10 N; HAP = 116, 124 and 138 MPa; R_a_(PBI) = 1.41 ± 0.43 μm; R_a_(PEEK-PBI) = 1.27 ± 0.18 μm; R_a_(PEEK) = 0.79 ± 0.15 μm; R_a_(Steel) < 0.01 μm).*

In [33] Zhang, G., et al. analyzed the effect of the amorphous structure of a PEEK film on its tribological properties. It was shown that there is no relative movement between a ball and the amorphous PEEK surface during the stick-slip sliding process due to the adhesion force in the interface zone. As a result, the material accumulated ahead of the counterpart. During the stick stage, shear stresses were less than the critical level required for adhesion between the sliding parts, but it increased with time. At high levels of the applied load, the tribological properties of amorphous PEEK could be closely related to its viscoelastic behavior, while the dominant factor was the ‘ironing’ effect at low load-speed levels. The change in the tribological properties of amorphous PEEK with an increase in sliding speed may be associated with the rising interfacial temperature. The critical load may be lower than that at which there is no pronounced viscous flow *(PEEK; B-o-D; T = 20 °C; RH = 70%; counterpart is the 100Cr6 steel ball with a diameter of 6 mm; R_a_ = 0.02 μm; L = 2 km; P = 1–9 N; V = 0.2–1.4 m/s).*

### 2.2. Reinforced HPP-Based Composites

Yamaguchi, T., et al. investigated wearing of PEEK-based composites loaded with particles of rice bran ceramics (RBC) under water lubrication (WL) conditions [34]. The ‘PEEK + RBC (10–40 wt.%)’ samples showed a decrease in both CoF and specific WR values over a wide range of the P·V conditions compared to those for neat PEEK. The authors suggested that the reason was the hydrodynamic lubrication effect. *(PEEK; B-o-D; R_a_ = 0.14 µm; counterpart is the SUS 304 steel ball with a diameter of 8 mm; WL; V = 0.1–2.0 m/s; P = 0.98–9.80 N)* Dong, F., et al. assessed the friction and wear behavior of neat PI and ‘PI + CF’ reinforced composites at various temperatures [35]. Loading with CFs significantly improved wear resistance over the entire temperature range, and CoF levels were highly dependent on the sliding contact temperature. It was found that the CF lubricity appeared only at high temperatures of 180–260 °C, which could be explained by their graphitization and subsequent TF formation on the worn surfaces with excellent lubricity *(PI; B-o-D; R_a_ = 0.03 μm; counterpart is the GCr15 steel ball with a diameter of 3 mm; R_a_ = 0.02 μm; V = 0.3 m/s (573 r/min); P = 5 N; t = 30 min).*

Lv, M., et al. reported the tribological behavior of PI-based composites reinforced with CFs and AFs under simulated space irradiation conditions and during the ‘start-stop’ friction process [36]. Loading with CFs and AFs contributed to lower CoF values and improved wear resistance of the composites, especially in the case of CFs. After UV irradiation, the composites maintained low both CoF and WR levels. This was attributed to continuous TF formation on the counterpart. It should be noted that tribological testing in the ‘start-stop’ mode resulted in rising WR values *(PI; B-o-D; RT; vacuum of 10^−4^ Pa; counterpart is the GCrl5 steel ball with a diameter of 3.175 mm; R_a_ = n/d; V = 0.126 m/s; RR = 6 mm; P = 1 N).*

Loading with thermally conducting fillers increases the thermal conductivity and the mechanical strength of polymer composites. Debashis Puhan et al. reviewed the effect of SCFs on the TF nature and the tribological performance of the ‘50% PEEK + 50% PBI’ composite upon friction on the steel counterpart at temperatures up to 300 °C [37]. The TF contained materials mostly related to SCFs. As a result, the CoF level reduced compared to those for neat PEEK and PBP, especially near the (TgPEEK) glass transition temperature of PEEK, when the TFs were relatively thick. At 300 °C, the ‘PBP + SCF’ TF became thin, possibly due to abrasion by SCFs dislodged from the matrix. Below the T_g_ PEEK level, the ‘50% PEEK + 50% PBI’ composite was characterized by greater WR values than that for neat PEEK, while thick TFs formed above this temperature and wear resistance of the composite was enhanced *(PEEK-PBI; B-o-D; counterpart is the AISI 52100 steel ball with a diameter of 6 mm; R_a_ = n/d; DSF; P = 10 N; V = 1 and 2 m/s; T = 25, 100, 145, 200 and 300 °C; L = 3.6 km).*

In [38] Jacobs, O., et al. analyzed the effect of the counterpart material (the 100Cr6 and X5CrNi18-10 steels, alumina, and a bronze) on WR levels of PEEK-based composites loaded with CFs, GF, PTFE, and Gr at low P·V values under DSF and in an aqueous medium. At the DSF conditions, the lowest WR level was typical for stainless steel, while the best results were observed for alumina in the aqueous environment. The ‘GF + PTFE’ composite showed the greatest wear resistance. CFs are preferred for friction in water as they react very susceptibly with this environment. As a rule, friction in the aquatic medium contributes to increased WR values. It has been shown that the filler material can change the WR levels by several orders of magnitude at the minimum achieved level of ~10^−8^ mm^3^/Nm. Correctly choosing the counterpart materials could more effectively improve wear resistance of the composites than thorough designing their compositions *(B-o-P; unidirectional sliding; R_z_(100Cr6) = 0.15 µm; R_z_(X5CrNi18-10) = 0.31 µm; R_z_(Al_2_O_3_) = 0.46 µm; R_z_(bronze) = 0.33 µm; P = 30.0 N; P = 21.2 N; RF = 1 Hz; V = 28.2 mm/s; L = 6 km; CP = 3–27 MPa; P·V < 0.76 MPa m/s).*

Greco, A.C., et al. tested both neat PEEK and composites reinforced with short randomly oriented fibers (SROF) as well as long woven fibers (LWF) at high sliding speeds [39]. The presence (and morphology) of such fibers significantly affected the wear mechanisms under these test conditions. CoF levels decreased with increasing the load for all tested materials. The ‘PEEK + LWF’ composite had the lowest CoF level (50% fewer than that after loading with SROF). The same was relevant to the ‘PEEK + LWF’ one, whose WR value was an order of magnitude lower than that of the ‘PEEK + SROF’ sample. In the case of high loads, the WR level of the ‘PEE + LWF’ composite increased due to fracture of fibers, the abrasive wear of the counterpart surface, and TF removal *(PEEK; B-o-D; counterpart is the 440c steel ball with a diameter of 4.76 mm; ball-on-disc unidirectional sliding; R_a_ = n/d; P = 0.73, 2.50, 5.00 and 10.00 N; MHS = 50–600 MPa).*

When designing PI-based composites for high-temperature applications, 3-APTES and its modification with lanthanum (La) salt can be used for improving the interface properties. As an example, Yu, L., et al. investigated ‘PI + poly-p-phenylenebenzobisoxazole (PBO)’ samples [40]. They reported that the PBO fiber surface modification with La salt was the most efficient. CoF levels decreased for all studied composites with rising load-speed parameters at both T = 130 and 260 °C, while WR values increased in this case. In contrast to T = 130 °C, wear resistance of the unmodified composite was highly dependent on both load and speed parameters at T = 260 °C. For comparison, the modified one was less affected. Under the same tribological test parameters, the ‘PI + PBO-La’ composite showed the lowest specific WR and CoF levels *(PI; B-o-D; counterpart is a metal ball of 89 HRA; R_a_ = 0.1 μm; T = 130 and 260 °C; t = 60 min; P = 3–12 N; V = 0.25–1.00 m/s)*

Li, E.Z., et al. reported the effect of applied load and sliding duration on both CoF and WR levels of the ‘PEEK + 30 wt.% GF’ composite [41]. It was shown that these characteristics gradually increased and remained stable with both rising tribological test parameters. The composite possessed a markedly higher wear resistance compared to that for neat PEEK. GF were extruded from the composite rather than pulverized into it. The thermal decomposition temperature increased by 75 °C for the composite compared to that for neat PEEK. *(PEEK; B-o-D; R_a_ = n/d; RT; P = 100–400 N; t = 30 and 120 min).*

### 2.3. Solid-Lubricant Fillers

After loading with SLs, TF formation is determined by a mechanism providing an increase in the polymer wear resistance (primarily due to a decrease in CoF values). Duan, C., et al. developed an effective approach for improving the high-temperature wear resistance of thermoset PI (TPI) by loading it with g-C_3_N_4_ (0.5–5.0 wt.%) [42]. In a range of room temperature up to 350 °C, wear resistance of these composites improved greatly and reached 7.29 × 10^−7^ mm^3^/Nm (T = 350 °C at the g-C_3_N_4_ content of 5 wt.%). The observed effect was achieved due to TF formation, containing g-C_3_N_4_ with both high elastic modulus and hardness levels. This enabled the composites to effectively redistribute loads on TPI and prevent resin from abrading at the contact surfaces *(PI; B-o-P; R_a_(TPI) = 0.093 µm; counterpart is the GCr15 steel ring with a diameter of 3 mm; P = 5 N; V = 0.1 m/s; L = 1 km; t = 166 min; RT, 100, 200, 250, 300 and 350 °C).*

The authors of this review initially did not plan to analyze PTFE-based composites, but one such paper is of particular interest. In [43] Zhao, Y., et al. studied the effect of loading PTFE with silicon dioxide (SiO_2_-PI) in amounts of 0–15 wt.% and porous PI (0–15 wt.%) on the tribological properties of the composites. Compared to loading with neat PI, the addition of SiO_2_-PI and porous PI resulted in loose structures of the PTFE matrices and uneven worn composite surfaces. This promoted the formation of smooth and uniform TFs. In contrast to loading PTFE with SiO_2_-PI, the addition of SiO_2_ nanoparticles into the ‘PTFE + PI’ compounds induced the formation of rough and uniform TFs, which were detrimental to the tribological performance of the ‘PTFE + PI + SiO_2_′ composites *(PI, B-o-F, R_a_ = n/d; P = 200 N; stroke length = 15 mm; frequency = 2 Hz; t = 120 min; counterpart is the GCr15 steel ball with a diameter of 9.5 mm).*

Concerning hybrid HPP-based composites, their loading with SLs and reinforcing fibers is a typical solution for improving functional properties. Song, J. investigated insulated PI-based ones filled with SGF, PTFE, SiO_2_ and PPL [44]. When replacing CFs and Gr with GF and PTFE, both CoF and WR parameters were reduced. The achieved high tribological characteristics were interpreted as a synergistic effect of the simultaneous loading of the PI matrix with GF, PTFE, SiO_2_, and PPL *(PI; B-o-D; counterpart is the Si3N4 ball with a diameter of 4 mm; R_a_ = n/d; V = 0.063 m/s; P = 3 N; t = 6 h.*

In addition to the production of bulk polymer composites, the formation of coatings is a perspective. Demian, C., et al. developed thermoset PI-based composite coatings on aluminum substrates [45]. PTFE and SiC (up to 5 wt.%) fillers were added into neat PI. It was shown that loading with PTFE significantly reduced CoF levels of the ‘PI + PTFE + SiC’ composite coatings, as PTFE particles contributed greatly to the reduction in the total surface energy. The ‘PI + 20% PTFE + 5% SiC’ composite coating was characterized by a low CoF value with a slightly decreased WR level as well as satisfactory hardness compared to those for other ‘PI + PTFE + SiC’ samples. *(PI; B-o-D; counterpart is the 100C6 steel ball with a diameter of 6 mm; R_a_ = 0.02 μm; V = 0.4–0.8 m/s; P = 5 N)* Zhang, G., et al. studied two PEEK-based coatings deposited on aluminum substrates by flame spraying and printing [46]. After annealing at 260 °C for 30 min, an initially amorphous coating obtained a semicrystalline structure, possessing both lower CoF and WR levels. The addition of SiC microparticles and Gr into the PEEK-based coatings significantly improved their wear resistance *(PEEK; B-o-D; counterpart is the 100Cr6 steel ball with the diameter of 6 mm; R_a_ = 0.02 μm; P = 5 N; V = 0.2 m/s; L = 2 km).*

Lal, B., et al. analyzed a series of PEEK-based composites loaded with PTFE particles (0–20 wt.%) [47]. The addition of the latter enabled enhanced ultimate load values, eliminated the stick-slip tendency, and greatly decreased both CoF and specific WR parameters. The lowest CoF value was recorded at 15% PTFE. At T = 100 °C, the CoF and WR levels increased slightly for all studied composites. On the other hand, the composites showed lower wear resistance than neat PEEK in the abrasion tribilogical mode *(PEEK, B-o-P; counterpart is the 100Cr6 steel ball with a diameter of 10 mm; R_a_ = n/d; frequency = 50 Hz; RT; L = 360 m; T = 100 °C; P = 50 N).*

### 2.4. Nanofillers

As noted above, the role of nanoparticles in HPP-based composites for tribological applications narrows to (1) reinforcing the polymer matrix; (2) (solid) lubricating (with correct compositions); (3) stimulating TF fixation on counterparts. The most ‘popular’ types of nanofillers were also listed. Results of a number of papers devoted to the point composite-counterpart contacts are summarized below.

Zhao, Y., et al. fabricated ‘PI + nano-SiO_2_’ (0–25 wt.%) composites via in situ polymerization [48]. First, CoF levels of the composites decreased by 6.8% with an increase in the nano-SiO_2_ content from 0 to 5 wt.%. Then, they enhanced CoF levels by 11% with raising the content up to 25 wt.%. At the nano-SiO_2_ contents of 5–10 wt.%, WR values of the composites were greater than that for neat PI. Formed TFs delaminated easily, and nano-SiO_2_ particles tended to be accumulated on the friction surfaces. These factors reduced wear resistance of the composites (*PI; B-o-D; R_a_ = n/d; RT; t = 1.5 h; V = 0.04 and 0.08 m/s; P = 5 and 10 N; counterpart is the GCr15 ball with a diameter of 3 mm*). Zhou S., et al. investigated PI-based composite coatings composed of CNTs and fluorinated graphene (FG), in which both hybrid and blend phases were mixed via solution doping [49]. A significant increase in the mechanical properties was accompanied by a decrease in WR levels by 61%. For the ‘PI + CNT + FG’ composite coating, the CNT strengthening effect and the FG lubrication characteristics were fully manifested in the blend phase *(PI; B-o-D; R_a_ = n/d; SF = 2 Hz; DSF; t = 30 min; P = 5 N; counterpart is the GCr15 steel ball with the diameter of 3 mm).*

In [50] Chen, B., et al. prepared multiscale ‘CF + CNT’ PI-based composites using a chemical method. The composites possessed many functional groups and increased roughness. CoF and WR values of the ‘PI + CF + CNT’ composites were 0.213 and 1.79 × 10^−6^ mm^3^/Nm, i.e., decreased by 22% and 72%, respectively, compared to those for neat PI. Also, both CoF and WR levels decreased by raising the load-speed product. Thus, CNTs stretched into the PI matrix on the hybrid ‘CF + CNT’ surface, which had a reinforcing effect even at high load-speed parameters *(B-o-F; RS; counterpart is the GCr15 ball; R_a_ = n/d; DSF; t = 30 min; P = 1.5–4.5 N; LRV; V = 0.05, 0.083 and 0.116 m/s).*

Nanofillers are made in complex shapes to improve their adhesion. Yuan, H., et al. loaded PI with MoS_2_ nanoflowers (0.5–1.5 wt.%) through grafting them onto the surfaces of hollow CNFs (HCNFs) [51]. The ‘PI + MoS_2_ + HCNF’ composite coatings showed high wear resistance under WL conditions (with a filler content of 0.5 wt.%, the WR reduction was 72.5%) and in liquid paraffin oil (with a filler content of 1.5 wt.%, the WR value decreased by 56.0%) *(PI; B-o-P; the counterpart is the GCr15 steel ball with a diameter of 6 mm; R_a_=; RT; RH = 25–30%; HCP = 112 MPa; P = 20 N; V = 1.2 cm/s (20 Hz); t = 30 min).* Graphitic carbon nitride (g-C_3_N_4_) is being extensively investigated by many researchers as a nanofiller for improving the tribological properties of PI-based composites. For example, Zhu, L., et al. showed [52] that its contents from 0.5 up to 2.0 wt.% had a decisive influence on the tribological properties of the ‘PI + g-C_3_N_4_’ composites. CoF kinetics gradually decreased with raising the g-C_3_N_4_ filler concentration but increased above 10 wt.%. At its low contents, the wear type was adhesive, while it changed to abrasive with excessive g-C_3_N_4_ levels *(PI; B-o-D; R_a_ = n/d; DSF; reciprocating testing; P = 2, 4 and 50 N; counterpart is the GCr15 ball with the diameter of 6 mm; t = 10 min; V = 0.42 m/s; RT; RH = 40%).*

Chen, B., et al. designed a novel hybrid PI-based composite with micro-CFs and hexagonal MoS_2_ nanosheets (0.5–20.0 wt.%) via a one-step hydrothermal method [53]. The hybrid simultaneously exerted both lubricating and strengthening effects on the PI matrix. This contributed to transferring stresses from the matrix to CFs during friction and wear. The ‘PI + CF + MoS_2_’ composite showed advanced tribological properties. CoF and WR values were equal to only 0.24 and 2.01 × 10^−6^ mm^3^/Nm, respectively, which were lower than those for neat PI, as well as for both ‘PI + CF’ and ‘PI + MoS_2_’ composites. Additionally, self-lubricating MoS_2_ nanosheets gradually exposed on the worn surfaces, making it easier to slide *(PI, B-o-F; R_a_ = n/d; P = 3 N; V = 0.083 m/s; t = 30 min).*

**Figure 8:**
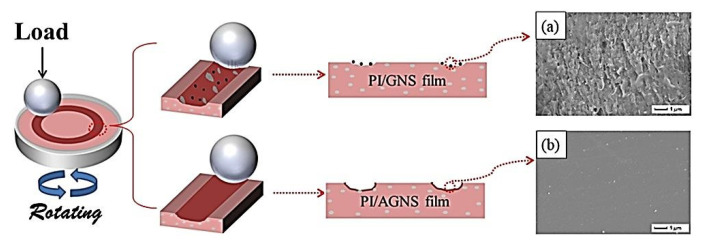
A model of the friction mechanism for the ‘PI + GNS’ and ‘PI + AGNS’ films. The inset is the SEM images of worn surface of (**a**) PI/GNS-1wt.% nanocomposite and (**b**) PI/AGNS-1wt.% nanocomposite. Figure is reproduced with permission from reference [54].

Min, C., et al. fabricated PI-based nanocomposites using different contents of amine-functionalized graphene nanosheets (AGNS) via in situ polymerization [54]. In contrast to neat PI and ‘PI + GNS’ composites, the ‘PI + 0.5 wt.% AGNS’ sample was characterized by both reduced CoF and specific WR (by 41.9% and 72.6%, respectively. Under DSF conditions, this effect was provided by strong interfacial adhesion and good compatibility between AGNS and PI (Figure 8). In addition, the ‘PI + AGNS’ nanocomposite showed excellent wear resistance in seawater at CoF = 0.16 and WR = 1.68·10^−4^ mm^3^/Nm *(PI, B-o-D; R_a_ = n/d; DSF, SWL; V = 300 r/min; t = 30 min; P = 5 N; the counterpart is the GCr15 steel ball with a diameter of 4 mm).*

**Figure 9:**
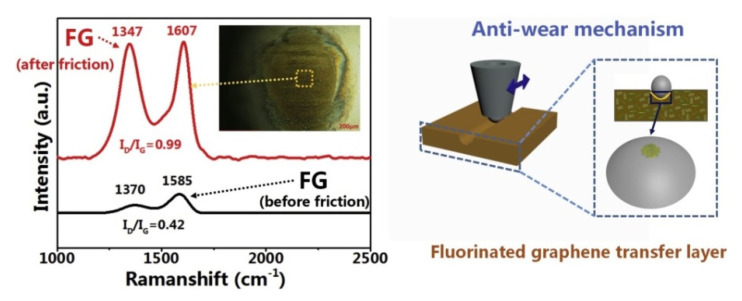
A schematic presentation of the tribological mechanism for the ‘PI + FG’ nanocomposites. Figure is reproduced with permission from reference [55].

Zhou, S., et al. investigated ‘PI + FG’ nanocomposite films (FG is fluorinated graphene in amounts of 0.5–2.0 wt.%) under different friction conditions [55]. CoF levels of the nanocomposites decreased slightly compared to that for neat PI. During the DSF tribological test, a WR value reduced by 51.2% for the composite with the optimum filler content of 0.5%. However, the WR levels decreased significantly for all samples due to the softening effect of water on the ‘PI + FG’ mixture, indicating that they were unsuitable for operation in aqueous environments. The presence of products of tribochemical reactions on the wear track surfaces provided an antiwear effect and reduced the CoF values (Figure 9). The FG layer covered the steel counterpart surface and formed a dense TF. Also, it reduced the CoF values and improved wear resistance *(PI; B-o-D; the counterpart is the GCr15 steel ball with a diameter of 3 mm; R_a_ = n/d; frequency = 5 Hz; P = 10 N; t = 30 min; DSF, SLF).* Min, C., et al. designed the ‘PI + 0.5 wt.% FGO’ nanocomposite [56]. Due to covalent bonds, the great FGO to PI interfacial adhesion increased their mechanical strength and wear resistance. At 0.5 wt.% FGO nanosheets, both CoF and WR parameters were reduced by 33.1% and 80.8%, respectively, compared to neat PI under the DSF conditions. In SWL, a CoF level was equal to 0.22, while the WR value of 0.45·10^−4^ mm^3^/Nm was registered *(PI, B-o-D; R_a_ = n/d; DSF and SWL; P = 5 N; V = 300 r/min; t = 30 min).*

**Figure 10:**
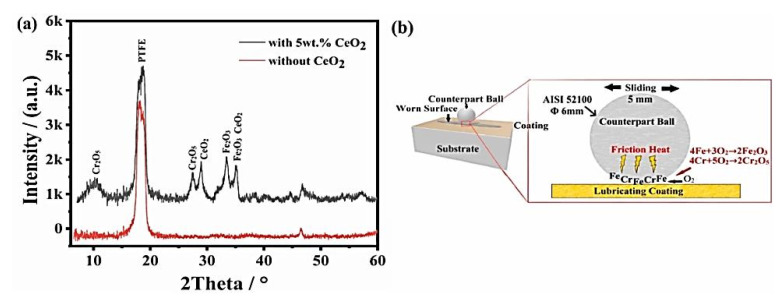
(**a**) Results of XRD analysis of the worn ‘PAI + PTFE’ coating surface and (**b**) a scheme of the tribochemical reactions. Figure is reproduced with permission from reference [57].

In [57] Zhao, Z., et al. used CeO_2_ nanoparticles (about 80–110 nm, 0–9 wt.%, prepared via in situ synthesis) for strengthening ‘PAI + PTFE’ coatings. At a concentration of CeO_2_ nanoparticles of 5 wt.%, the worn coating surface was the least damaged. During friction, substance transfer was observed, accompanied by tribochemical reactions. Due to TF formation, it was possible to exclude direct contact between the components of the mating materials (Figure 10), which improved their wear resistance. It was concluded that the ‘PAI + PTFE’ coatings are more suitable for high sliding speeds at low loads *(PAI; B-o-F; R_a_ = n/d; LRF; Dist = 5 mm; P = 5 N; V = 10 cm/s; L = 400 m; RT; AH = 35–55%).*

**Figure 11:**
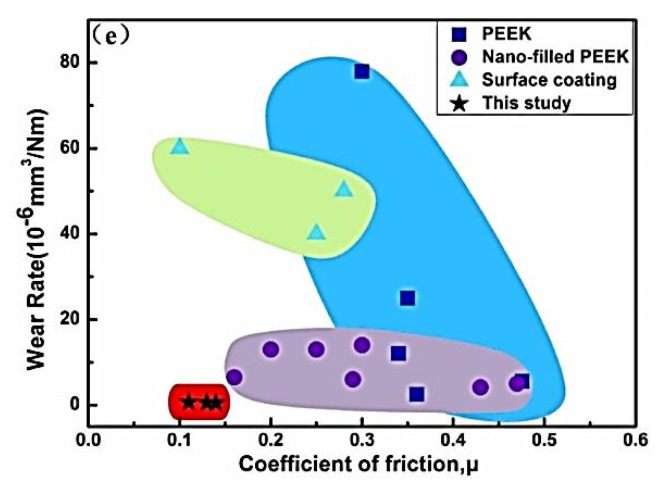
The WR vs. CoF diagram by Ashby (Zhang, 2010; Panin et al., 2021; Yan et al., 2020; Wang et al., 2017; Tharajak et al., 2015, 2017). Figure is reproduced with permission from reference [59].

Puértolas, J. A., et al. used the lubricant capability of graphene nanoplatelets (GNPs) to improve PEEK tribological properties [58]. Nanocomposites were fabricated by solvent-free melt-blending and injection molding at the filler contents of 1–10 wt.%. The CoF reduction was about 83% for the ‘PEEK + 10% GNP’ composite. This effect was associated with an increase in its hardness. At high GNP contents, the wear mechanism was fatigue, while the abrasive one dominated at the lowest concentrations. At the filling degrees of 3–5 wt.%, it was possible to reduce both CoF and WR at constant elastic modulus and strength levels *(PEEK; B-o-D; R_a_(PEEK) = 1 μm; counterpart is the alumina ball with a diameter of 6 mm; R_a_ = 0.050 ± 0.002 μm; WL; T = 37 °C; P = 5 N; HCP = 37 MPa; V = 0.05 m/s; L = 0.18, 2.16 and 4.52 km).* Due to the widespread use of PEEK in the field of artificial joint materials, Ting Wu et al. in situ integrated nano-ZnO particles (2.5–7.5 wt.%) in the PEEK powder surfaces by a one-step hydrothermal method [59]. As a result, their compressive strength reached 319 MPa, while the lowest WR level was 0.48 × 10^−6^ mm^3^/Nm for the ‘PEEK + 5% nano-ZnO’ composite, which was 68% lower than that for neat PEEK, according to Figure 11 *(PEEK; P-o-D; R_a_ = n/d; RLS; counterpart is the CoCrMo pin with a diameter of 10 mm; Dist = 10 mm; P = 30 N; V = 5 mm/s; t = 120 min; RT; 25% CSL).*

As a summary of this section, it is appropriate to cite the paper [60] by Guo, L., et al., that reviews papers on tribological test results of PEEK-based composites using the pin-on-disk scheme published in the last 20 years and provides new experimental data. In their research, PEEK was reinforced with CFs and loaded with Bi_2_O_3_ and SiO_2_ nanoparticles, as well as with conventional SLs (Gr and PTFE). SL particles improved wear resistance at low sliding speeds, especially with negligible loads. Chelation reactions between PTFE and the steel counterpart, as well as transfer of graphitic materials deriving Gr and CF debris were the main mechanisms driving TF growth. When sliding at a P = 1 MPa and speeds from 0.05 up to 0.20 m/s, additional loading with Bi_2_O_3_ nanoparticles significantly reduced WR levels of the CF-reinforced PEEK-based composite. At higher pressures or lower speeds, the effect of Bi_2_O_3_ nanoparticles was negligible. At low loads and relatively high speeds, releasing Bi_2_O_3_ nanoparticles on the friction surface improved TF load-bearing capability. The positive role of SiO_2_ nanoparticles was manifested at P = 30 MPa and V = 0.1 m/s. In the case of sliding at P = 30 MPa and V = 0.01 m/s, a great load and a relatively high speed contributed to the formation of strong TF reinforced with SiO_2_. The antiwear role of the SLs was the most pronounced at both low loads and speeds *(PEEK; P-o-D; counterparts are polymer pins and the GCr15 steel disk; R_a_ = 0.25 μm; DSF; RT; radius = 16.5 mm; V = 0.01–0.20 m/s; P = 1–30 MPa; t = 5 h).*

## 3. Line Contacts

### 3.1. Neat HPPs

C. Li and F. Yan compared the friction and wear characteristics of PTFE, UHMWPE and PI under DSF, blowing air to simulate sand-dust conditions [61]. Among the studied polymers, PI had the highest wear resistance in the DSF mode (WR = 0.2 × 10^−5^ mm^3^/Nm). However, under blowing air and sand-dust conditions, its WR values increased by 1.5–3.0 times. Blowing air resulted in reducing CoF levels of PI due to TF formation on a counterpart and raising its WR parameter. In doing so, blowing air significantly promoted the transfer of PI on the counterpart in contrast to the DSF conditions. PI exhibited the highest WR values because no TF was formed during the abrasive wear process *(PI; B-o-R; counterpart is the 1045 steel; R_a_ = 0.06–0.07 μm; RT; V = 0.424 m/s; P = 100 and 250 N).* In [62] the same authors investigated the dependence of PI wear resistance from applying loads under both DSF and simulated sand-dust conditions. With an increase in the load by more than 5 times, WR levels enhanced from 0.2 × 10^−5^ up to 0.6 × 10^−5^ mm^3^/Nm under the DSF conditions. However, it reduced to (0.6–0.8) × 10^−5^ mm^3^/Nm at the maximum load in another case. The higher hardness of PI and the fragmentation of abrasive particles under high loads were responsible to the WR reduction with raising the load *(PI; B-o-R; counterpart is the 1045 steel; R_a_ = 0.06–0.07 μm; RT; V = 0.424 m/s; P = 50–250 N).*

Y. Ma, et al. investigated the effects of the condensed-state structure on functional properties of both PPS and its fibers [63]. It was concluded that proper heat treatment of raw PPS fibers enables improved crystallinity degree and appropriate oxidative cross-linking. As a result, they exhibited better mechanical and tribological characteristics *(PPS; B-o-R; counterpart is the AISI 1045 steel; R_a_ = 0.4 μm; RT; V = 0.42 m/s; P = 100 N).*

### 3.2. Polymer-Polymer Composites

Chen, J., et al. evaluated the tribological properties of ‘PEEK + PEI + PES’ plastic alloys, varying contents of the fillers from 0 up to 100 wt.% [64]. Compared to neat PEEK, their specific WR levels increased from 0.7 × 10^−5^ up to (5–6) × 10^−5^ mm^3^/Nm. However, since both neat PEI and PES possessed high specific WR values of 2.5 × 10^−4^ and 3.1 × 10^−4^ mm^3^/Nm, respectively, loading with PEEK improved their wear resistance. A TF was formed on a counterpart only in the cases of neat PEEK and PEEK-based composites, but neat PEI and PES did not form any films on the steel counterpart surface *(PEEK; PE; PES; B-o-R; counterpart is the 30CrMnSiA steel ring; R_a_ = n/d; RT; V = 200 r/min; P = 200 N).*

In [65] Kim, S.S., et al. reported the effect of elastomeric polymers on the tribological and mechanical characteristics of PPS-based composites loaded with ethylene butyl acrylate (EBA). The addition of EBA (10 and 15 wt.%) reduced wear loss by a factor of five compared to that for neat PPS. The improvement of wear resistance was associated with the formation of more uniform TFs on the steel counterpart surfaces *(PPS; B-o-R; counterpart is a carbon steel; R_a_ = n/d; RT; V = 0.02–0.50 m/s; P = 200 N).* Shi, Y., et al. studied the influence of heat treatment of ‘PPS + polyamide 66 + PTFE’ composites fabricated by an injection molding process [66]. The ’56 wt.% PPS + 24 wt.% PA + 20 wt.% PTFE’ sample exhibited the best tribological properties after annealing at 150 °C for 1 h. The lowest CoF and specific WR values were 0.139 and 4.372 × 10^−6^ mm^3^/Nm, respectively, which were fewer by about 0.8 and 1830 times than those for neat PPS *(PPS; B-o-R; counterpart is a titanium alloy; R_a_ = 0.7–0.9 μm; RT; V = 0.42 m/s; P = 200 N).*

### 3.3. Fiber-Reinforced Two-Component Composites

Chen, B., et al. reported the effect of the addition of polyacrylonitrile (PAN) CFs with lengths of 28–56 μm in PEEK on the tribological behavior of the obtained composites [67]. The ‘PEEK + CF’ samples possessed better friction and wear properties under SWL than in pure water and without any lubrication. Under the SWL conditions, the composite with 10 vol.% CFs exhibited the highest wear resistance, whose WR level was 3.23 × 10^−7^ mm^3^/Nm at a load of 600 N. This result was caused by the fact that CFs could effectively redistribute the applied load between the contacted surfaces and, consequently, protect the matrix from accelerated wear *(PEEK; B-o-R; counterpart is the 316 steel; R_a_ = 0.1 μm; RT; V = 0.5 and 1.0 m/s; P = 200 and 600 N).*

In [68] Gebhard, A., et al. investigated the issue of increased WR values for aqueous lubricated ‘PEEK + SCF’ composites due to galvanic fiber corrosion. When an electric bias voltage was applied to the samples loaded with 30 vol.% SCFs, they were characterized by extensive fiber corrosion that greatly increased their WR levels. Since the composites containing only 10 vol.% SCFs are electric insulators, they were treated to be immune to the mentioned degradation process *(PEEK; B-o-R; counterpart is the X10CrNiMoTi1810 steel; R_a_ = 0.1–0.2 µm; RT; V = 1 m/s; CP = 1.5 MPa.)* Oyamada, T., et al. reported the effect of gas environments on the tribological properties of ‘PEEK + CF’ composites [69]. The obtained results showed that CoF values decreased from 0.25 down to 0.17 when the humidity changed from 40% down to 2%. The CoF levels of the ‘PEEK + CF’ composites dropped below 0.1 in low-humidity low-oxygen environments such as dry nitrogen, especially at cryogenic temperatures (the lowest CoF value was 0.03 at 113 K). At the same time, WR levels of the ‘PEEK + CF’ composites also decreased. The reason was the fact that TFs covered irregularities on the contact surfaces and reduced their roughness *(PEEK; B-o-R; R_a_ = 0.2 µm; counterpart is a steel; Block R_a_ = 0.4 µm; V = 2 mm/s; P = 490 N).*

In [70] Xu, H., et al. studied PPS-based composite coatings deposited by flame spraying and reinforced with CFs (75 μm). They possessed high wear resistance only under WL conditions. In this case, iron was transferred from a steel ring on the worn surface of the composite coating and CFs were exposed to dynamic loads. This improved their wear resistance *(PPS; B-o-R; counterpart is a steel; R_a_ = n/d; V = 0.43 m/s; P = 200 N).* Zhao, G., et al. described the effect of the addition of CF, GF and AF fibers on wear resistance of PI-based composites under both DSF and three-body abrasive conditions [71]. It was shown that the composites with inorganic fibers (CFs and GFs) possessed the highest wear resistance when slid on a steel ring under DSF conditions. In this case, WR levels were (2–3) × 10^−6^ mm^3^/Nm. Under the three-body abrasion conditions, the composites had worse wear resistance than that of neat PI. The main reason was poor interfacial adhesion between fibers and the polymer matrix *(PI; B-o-R; counterpart is a steel; R_a_ = n/d; V = 1.0 and 2.4 m/s; P = 30–130 N).* The same authors further developed this theme in [72] by varying the tribological test temperatures from the room level up to 200 °C. With the rise, WR levels enhanced as well. The highest wear resistance was observed for the composite loaded with GFs. At T = 200 °C, the WR value was 1.2 × 10^−7^ mm^3^/Nm *(PI; B-o-R; counterpart is the GCrl5 steel; R_a_ = 0.1 μm; V = 1 m/s; P = 30 N).*

In [73] Zhang, X., et al. reported the influence of contents of SBF (3–4 mm long) on the tribological properties of PI-based composites. The best combination of CoF and WR values (0.27 and 4 × 10^−5^ mm^3^/Nm, respectively) was found with the SBF content of 10 wt.%. A TF, formed on the counterpart surface, contributed to the reduction of these parameters *(PI; B-o-R; counterpart is the GCrl5 steel; R_a_ = 0.2 μm; V = 43.1–86.2 cm/s; P = 200–500 N).* Treatment of fibers with acid solutions is typically considered as an effective way to activate their surface. In [74] Zhang, X., et al. confirmed this fact on an example of CF and HNO_3_ plus diamine modification followed by their loading into the PI-based matrix. For untreated fibers, a WR value was 7.0 × 10^−6^ mm^3^/Nm, while this parameter reduced to 4.4 × 10^−6^ mm^3^/Nm after the mentioned procedure. This improvement was related to enhanced interface adhesive strength *(PI; B-o-R; counterpart is the GCrl5 steel; R_a_ = 0.2–0.3 µm; V = 43.1 cm/s; P = 200 N).*

In addition, Zhang, X., et al. modified PAN-based CFs by strong HNO_3_ oxidation and then loaded them into PI-based composites [75]. The CF weight percent was 70%. Under the tribological test parameters similar to the previously mentioned paper [72] a WR value was 3.7 × 10^−6^ mm^3^/Nm for the ‘CF + PI’ composite. SEM and XPS studies of the CF surface showed that it had become rougher and the oxygen concentration increased greatly. These facts resulted in rising adhesion between fibers and the PI matrix, reducing CoF levels, and improving the tribological properties *(PI; B-o-R; counterpart is the GCrl5 steel; R_a_ = 0.1 μm; V = 1 m/s; P = 30 N).*

### 3.4. Three-Component Reinforced Composites

**Figure 12:**
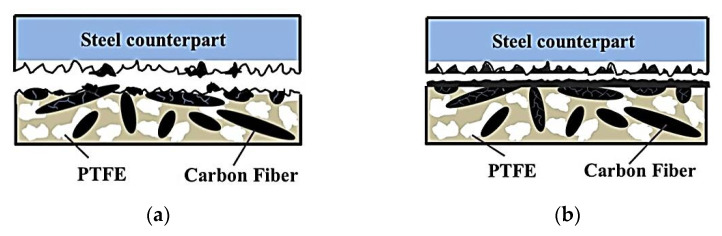
Schematic diagrams of TF formation for the ‘PPS + PTFE + SCF’ composites at loads of 200 (**a**) and 500 (**b**) N. Figure reproduced with permission from reference [76].

Luo, W., et al. investigated the effect of the test conditions and the PTFE and SCF contents on the tribological properties of PPS-based composites [76]. The sample loaded with 15 vol.% SCFs exhibited the highest WR level of 5.2 × 10^−6^ mm^3^/Nm and the lowest CoF value of 0.085, which were 88% and 47% fewer than those for the ‘mono-PPS + PTFE’ composite. The worn surfaces showed that the wear mechanism of the ‘PPS + PTFE’ composites transformed from adhesive to abrasive due to loading with SCFs (Figure 12) *(PPS; B-o-R; counterpart is a steel; R_a_ = 0.7–0.9 μm; RT; V = 0.42 m/s; P = 200 and 500 N).*

**Figure 13:**
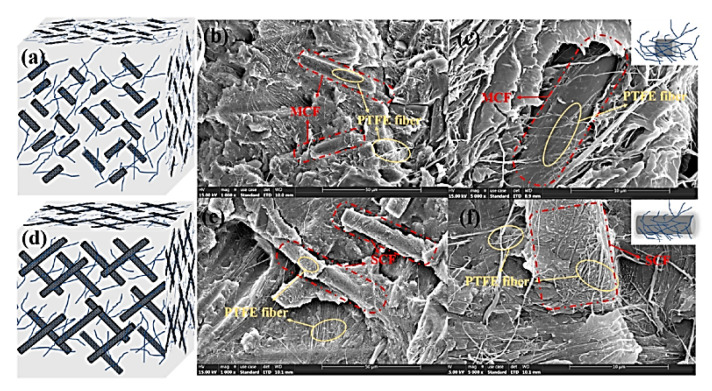
Schematic of ‘PPS/PTFE/MCF’ composites (**a**); structural morphology of ‘PPS/PTFE/MCF’ composites and enlarged view (**b**,**c**); schematic of ‘PPS/PTFE/SCF’ composites (**d**); structural morphology of ‘PPS/PTFE/SCF’ composites and enlarged view (**e**,**f**). Figure is reproduced with permission from reference [77].

Shi, Y., et al. reported the effect of contents of multiscale CF and PTFE fibers on the tribological properties of PPS-based composites [77]. The sample loaded with SCFs possessed better characteristics compared to those for the MCF-filled composite. The optimal tribological properties were shown by the ‘PPS + 16 wt.% PTFE + 20 wt.% SCF’ sample that was characterized by the minimum CoF and WR values (0.103 and 3.79 × 10^−5^ mm^3^/Nm, respectively) due to the effective network structure inside the composites (Figure 13). An investigation of TFs and the worn surfaces of the composites showed that the wear mechanism gave priority to adhesive type accompanied by abrasive damages *(PPS; B-o-R; counterpart is a titanium alloy; R_a_ = 0.7–0.9 μm; RT; V = 0.42 m/s; P = 200 N).*

Zhang, X., et al. studied wear resistance of ‘PI + SBF’ composites with the addition of MoS_2_ and Gr under various load-speed conditions [78]. The best combination of both CoF and WR parameters was found at the optimal MoS_2_ (40 vol.%) and Gr (35 vol.%) contents. With rising P·V product, the CoF and WR values reduced. At V = 86.2 cm/s and P = 500 N, the composites showed the CoF = 0.07 and WR = 2.2 × 10^−7^ mm^3^/Nm *(PI; B-o-R; counterpart is the GCr15 steel; R_a_ = 0.2 μm; RT; V = 43.1–86.2 cm/s, P = 200–500 N).* In [79] Li, J. reported the effect of SGF and Gr powders on the tribological properties of PI-based composites. Loading with Gr significantly improved wear resistance of the samples. At a Gr content of 30 vol.%, a CoF level of 0.19 and a WR value of 0.5 × 10^−6^ mm^3^/Nm were registered under a load of 300 N and a speed of 42 cm/s *(PI; B-o-R; counterpart is the GCr15 steel; R_a_ = 0.1–0.2 μm; RT; V = 42.0 and 86.2 cm/s; P = 200–500 N).*

### 3.5. Multi-Component Reinforced Composites

Zhang, Z., et al. investigated the relation of the tribological properties of PEEK-based composites with contents of PTFE, Gr (5–15 vol.%) and SCFs (10–25 vol.%) as fillers [80]. The lowest WR level of 4.1 × 10^−7^ mm^3^/Nm was revealed for the ‘PEEK + 10% PTFE + 10% Gr + 20% SCF’ sample. This parameter exhibited a slight dependence on the material composition, elastic modulus, and density and impact resistance opposite to its flexural properties and toughness which were not so influential *(PEEK; B-o-R; counterpart is the 100Cr6 steel; R_a_ = 0.1 μm; RT; V = 1 m/s; CP = 1 MPa).*

Zhang, G., et al. showed the effects of fiber orientations and nominal contact pressures on the tribological properties of a multicomponent PEEK-based composite loaded with SCFs, PTFE, and Gr (10 vol.% for each) [81]. The fiber orientations were parallel, nonparallel, and normal relative to the sliding direction. At low loads (from 1 up to 2 MPa), no distinct difference in WR levels was observed for all studied patterns. By raising the load up to 3–5 MPa, the WR values were remarkably lower than those for the normal and parallel ones. As an example, a WR level of 9 × 10^−7^ mm^3^/Nm and a CoF value of 0.27 were determined for the sample with the nonparallel orientation *(PEEK; B-o-R; counterpart is the 100Cr6 steel; R_a_ = 0.3 μm; RT; V = 1 m/s; CP = 1–5 MPa).* In [82] Rasheva, Z., et al. investigated the influence of filler contents, fiber orientations, and nominal contact pressures on the tribological properties of ‘PEEK + PTFE + Gr + SCF’ composites. At the total PTFE and Gr level of 20 vol.% with 15 vol.% SCFs, the most consistent tribological behavior was noted, and uniformly worn surfaces were found, regardless of the fiber orientations and the applied loads. With the parallel fiber orientation, WR levels were equal to 0.413 × 10^−6^ mm^3^/Nm at P = 1 MPa and 0.420 × 10^−6^ mm^3^/Nm at P = 4 MPa, respectively. For the fiber orientation perpendicular to the sliding direction, the WR values were assessed as 0.424 × 10^−6^ and 0.468 × 10^−6^ mm^3^/Nm at the abovementioned loads, respectively *(PEEK; B-o-R; counterpart is the 100Cr6 steel; R_a_ = 0.3 μm; RT; V = 1 m/s, CP = 1–4 MPa).*

In [83] Zhang, X.-R., et al. studied multicomponent PI-based composites filled with SCFs, as well as micro-SiO_2_ and Gr particles. Both the lowest WR and CoF levels of 0.85 × 10^−6^ mm^3^/Nm and 0.18 were found, respectively, for the ‘PI + 10% Gr-10% SCF-3% SiO_2_’ composite at P = 200 N and V = 43 cm/s. It was also shown that the composites exhibited better tribological properties under higher P·V products *(PI; B-o-R; counterpart is the GCr15 steel; R_a_ =0.3 μm; RT; V = 43.1 and 86.2 cm/s, P = 200–700 N).*

### 3.6. Two-Component Nanocomposites

Lv, X., et al. developed PEEK-based implantable biomaterials with MoS_2_ nanosheets for applications in artificial joints [84]. The composite filled with 8 wt.% nanosheets had the highest wear resistance. In this case, a WR level was lower by more than three times compared to that for neat PEEK in both dry- and water-sliding contacts *(PEEK; B-o-R; counterpart is a steel; R_a_ = n/d; RT; V = 200 r/min; P = 200 N).* In [85] Zhang, L., et al. reported the effect of loading with hydroxylated g-C_3_N_4_ on the tribological properties of PEEK-based composites. It was shown that this filler possessed strengthened interlayer H-bonds and a denser stacking structure, which improved wear resistance. For the ‘PEEK + 10 vol.% h-CN’ composite, a WR value of 4 × 10^−7^ mm^3^/Nm was measured. At the same time, g-C_3_N_4_ transferred onto a counterpart and caused the generation of a load-carrying TF *(PEEK; B-o-R; counterpart is the GCr15 steel; R_a_ = 0.2–0.3 μm; RT; V = 1 m/s; P = 50 N).* The same authors investigated the addition of 2D g-C_3_N_4_ nanosheets (CNNS) on the tribological properties of PEEK-based composites under both boundary and mixed lubrication conditions [86]. It was shown that loading with CNNS resulted in a decrease in crystallinity and an increase in hardness, as well as a better wettability of PAO4 oil to the composites. Under the mixed lubrication conditions, the composite with 1 vol.% CNNS had a WR level of 1.5 × 10^−7^ mm^3^/Nm, which is 62% lower than that for neat PEEK. However, further raising the fraction of CNNS from 1 up to 2 vol.% gave rise to higher CoF and WR parameters *(PEEK; B-o-R; counterpart is a steel; R_a_ = 0.1–0.2 µm; RT; V = 0.03–0.80 m/s, P = 400 N).*

Zhang, G., et al. studied the influence of nano-SiO_2_ (0.5, 1.0, 2.0 and 4.0 vol.%) on the tribological properties of PEEK-based composites [87]. It was shown that these particles did not significantly affect CoF levels. The composite with 1 vol.% nano-SiO_2_ had the highest wear resistance at a WR value of 1 × 10^−5^ mm^3^/Nm. The nanocomposites were characterized by much smoother surfaces than neat PEEK. Reduced perpendicular strains of the PEEK matrix and decreased tangential plastic flows of the surface layer, involved in the friction process, were supposed to be important for changing the tribological behavior after loading with nano-SiO_2_ *(PEEK; B-o-R; counterpart is the 100Cr6 steel; R_a_ = 0.2 μm; RT; V = 1 m/s; CP = 4 MPa).*

In [88] Pan, S., et al. reported the effect of CNTs in amounts from 0.1 up to 0.9 wt.% on the tribological properties of PPS-based 3D-printed filaments. Their tensile and bending strength levels increased by 26% and 29%, respectively, compared to those for neat PPS. Under low loads, WR values reduced from 3.05 × 10^−5^ mm^3^/Nm for neat PPS down to 0.81 × 10^−5^ mm^3^/Nm of the ‘PPS + 0.7 wt.% CNT’ composite. At high loads, they decreased from 1.14 × 10^−3^ mm^3^/Nm for neat PPS down to 1.84 × 10^−5^ mm^3^/Nm for the ‘PPS + 0.9 wt.% CNT’ sample *(PPS; B-o-R; counterpart is a carbon steel; R_a_ = 0.3–0.4 μm; RT; V = 200 r/min, P = 100 and 196 N).* In addition, Cai, H., et al. showed the influence of CNTs (1–30 wt.%) on the functional properties of PI-based composites [89]. Their bending strength and microhardness rose with increasing filler content and reached stable values at 8 wt.% CNTs. Under DSF conditions, both CoF and wear volume loss levels decreased with enhancing the CNT content up to 8 wt.%. At P = 200 N and V = 43.1 cm/s, the wear volume loss was 2 mm^3^. A further increase in the CNT content did not cause a significant change in the properties of these nanocomposites *(PI; B-o-R; counterpart is a steel; R_a_ = 0.20–0.52 µm; RT; V = 43.1 and 86.2 cm/s; P = 50–196 N).*

**Figure 14:**
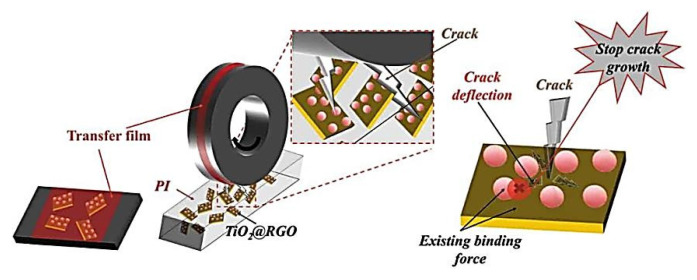
The friction mechanism of the ‘PI + TiO_2_@RGO’ composites. Figure is reproduced with permission from reference [91].

Cai, H., et al. presented results of loading PI with Al_2_O_3_ nanoparticles (1–12 wt.%) [90]. The nanocomposites containing from 3 up to 4 wt.% Al_2_O_3_ were characterized by the lowest WR levels under a relatively high load. At P = 200 N and V = 43.1 cm/s, the wear volume loss was 3 mm^3^ *(PI; B-o-R; counterpart is a steel; R_a_ = 0.20–0.52 µm; RT; V = 43.1 and 86.2 cm/s; P = 50–196 N).* In [91] Wu, J., et al. investigated the effect of decorated reduced graphene oxide (TiO_2_@RGO) in amounts from 0.25 up to 2.00 wt.% on the tribological properties of PI-based composites. At a high load of 300 N, a WR value decreased down to 2.64 × 10^−8^ mm^3^/Nm for the ‘PI + 0.5 wt.% TiO_2_@RGO’ sample. The obtained excellent mechanical and tribological properties were attributed to the structure of the composites and chemical bonds between RGO and TiO_2_, which were assumed to prevent the crack propagation under friction conditions and cause the WR reduction, according to Figure 14 *(PI; B-o-R; counterpart is a steel; R_a_ = 0.3–0.5 μm; RT; V = 42 cm/s, P = 100–300 N)*. Liu, H., et al. studied the influence of reduced graphene oxide (RGO) particles decorated with Fe_2_O_3_ ones [92]. Their incorporation in the PI matrix greatly enhanced the antiwear ability of the polymer films. The lowest WR value of 3.18 × 10^−8^ mm^3^/Nm, which was more than 28 times lower than that of neat PI, observed for the ‘PI + RGO + Fe_2_O_3_’ composite at a load of 300 N. In this case, the tribological wear mechanism was changed due to a synergetic effect of both Fe_2_O_3_ and RGO particles on the compressive and thermal properties of the composites. As a result, TF formation contributed to low WR levels under high loads *(PI; B-o-R; counterpart is a steel; R_a_ = 0.3–0.5 μm; RT; V = 42 cm/s; P = 100–300 N).*

In [93] Ruan, H., et al. loaded PI with ionic liquid functionalized graphene (ILFG). When the ILFG content was 0.4 wt.%, CoF and specific WR levels of the composite decreased by 38.2% and 25.0% compared to neat PI, respectively. A synergistic lubrication mechanism of ionic liquid and Gr caused TF formation under DSF conditions. The decomposition temperature of 5% weight loss (T5%) of the ‘IGPI + 0.4 wt.% ILFG’ composite reached 581.3 °C that was higher by 52.6 °C compared to that of neat PI. Also, the T_glass_ level increased by 14 °C *(PI; B-o-R; counterpart is the GCr15 steel; R_a_ = n/d; RT; V = 0.26–1.04 m/s, P = 15–30 N).*

The influence of a lubricating medium (deionized water or kerosene lubricant) on wear resistance of PI-based nanocomposites were reported in [94]. Also, the effect of their loading with graphene sheets (GNs) and perylene-3,4,9,10-tetracarboxylic acid dianhydride (PTCDA), at the GNs-to-PTCDA ratio of 1:1, on the tribological and mechanical performance was shown. A synergy was found between GN and PTCDA that resulted in low CoF levels and improved WR parameters for the composites in various mediums. Under DSF conditions, as well as in lubrication with deionized water and kerosene, the CoF values decreased by 41.1%, 70.0% and 35.7%, respectively *(PI; B-o-R; counterpart is a steel; R_a_ = n/d; RT; V = 0.26–1.03 m/s, P = 15–45 N).* Liu, H., et al. reported results of tribological tests under both DSF and WL conditions after loading PI with nano-TiO_2_ particles [95]. The highest WR improvement by 61% was observed with the optimum nano-TiO_2_ content of 3%, while the greatest CoF change of 60% was found after loading with nano-TiO_2_ particles in the amount of 9%. In contrast, under the WL conditions, the CoF levels of the nanocomposites were enhanced by raising the nano-TiO_2_ content. During the DSF tribological tests, the wear mechanism of the ‘PI + TiO_2_’ nanocomposites proceeded from fatigue wear to a combination of the fatigue and abrasive mechanisms with an increase in the nano-TiO_2_ fraction *(PI; B-o-R; counterpart is the GCr15 steel; R_a_ = 0.1 μm; RT; V = 0.515–2.060 m/s; P = 100–300 N).*

### 3.7. Three-Component Nanocomposites

Qiao, H.-B., et al. studied the effect of Al_2_O_3_ nano- or microparticles in an amount of 5 wt.% on the tribological properties of three-component ‘PEEK + 10 wt.% PTFE’ nanocomposites [96]. All investigated inclusions did not affect CoF levels of the samples. Without PTFE, a CoF level was 0.35 but it reduced to 0.25 after the PTFE addition regardless of the Al_2_O_3_ filler dimensions. The ‘PEEK + PTFE’ composite loaded with 15 nm Al_2_O_3_ particles possessed the lowest WR value of 5 × 10^−6^ mm^3^/Nm. For the composites loaded with both 90 and 500 nm Al_2_O_3_ inclusions, the increased WR value was associated with abrasive and adhesive wear types, respectively, on the surface of the PEEK-based composite compared to that loaded with 15 nm Al_2_O_3_ particles. The ability of such fillers to improve wear resistance was closely related to TF formation *(PEEK; B-o-R; counterpart is the AISI 1045 steel; R_a_ = n/d; RT; V = 42 cm/s; P = 196 N).* In [97] Wan, H., et al. showed the influence of 1–9 wt.% Ag nanoparticles on the tribological behavior of ‘PI + epoxy resin + PTFE’ bonded SL coatings. Their content of 5 wt.% enabled to reduce CoF values by 1.5 times under DSF conditions. The coatings were characterized by better tribological properties in RP-3 aviation kerosene (due to lubricating and cooling) than in the DSF process. Fatigue wear was observed regardless of the studied tribological conditions *(PI; B-o-R; counterpart is the GCr15 steel; R_a_ = 0.02 µm; RT; V = 42 and 84 cm/s; P = 50–150 N).*

Zhenhua L. reported the influence of titanium dioxide (TiO_2_, 5–20 vol.%) dispersion on the tribological properties of PI-based composites reinforced with CFs [98]. The highest wear resistance (WR = 0.3 × 10^−6^ mm^3^/Nm) was recorded for the ‘PI + 10 vol.% TiO_2_ + 20 vol.% CF’ sample. The incorporation of TiO_2_ caused a significant improvement in the matrix stiffness *(PI; B-o-R; GCr15 bearing steel; R_a_ = n/d; RT; V = 1.28–3.20 m/s; P = 200–1000 N).* In [99] Su, C., et al. investigated the effect of SiC nanoparticles (1–9 vol.%) on the tribological properties of PI-based composites loaded with CFc (plain weave, 65 vol.%). The bending strength increased by 25%, while WR levels decreased by 70% (equal to 5 × 10^−5^ mm^3^/Nm at a speed of 0.1 m/s) with a content of SiC nanoparticles of 5 vol.%. Enhancing tribological performance was caused by high mechanical and tribological properties of SiC nanoparticles and an improved bonding strength between CF and PI due to both high specific surface area and the surface activity of SiC inclusions. In addition, a thin and relatively uniform TF was formed on a steel counterpart *(PI; B-o-R; counterpart is the 440C steel; R_a_ = 0.1 μm; RT; V = 0.2–0.5 m/s; P = 600–1100 N; DSF).*

**Figure 15:**
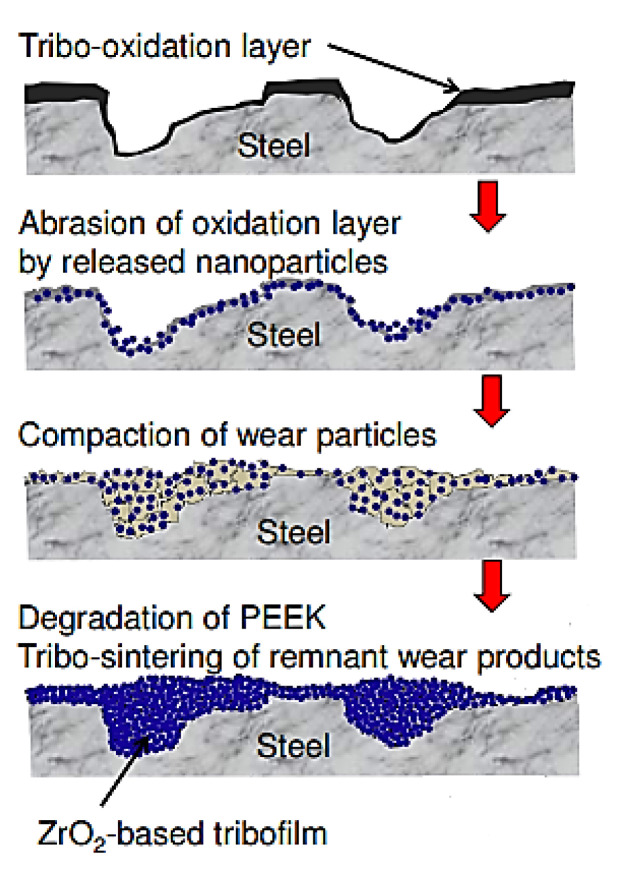
A schematic illustration of the effect of ZrO_2_ nanoparticles on TF formation. Figure is reproduced with permission from reference [100].

Guo, L., et al. investigated the tribological behavior of PEEK-based composites reinforced with CFs (10 vol.%) and loaded with various oxide nanoparticles, i.e., Bi_2_O_3_, CuO, SiO_2,_ and ZrO_2_ (1 vol.%) [100]. The addition of SiO_2_ and ZrO_2_ reduced both CoF and WR parameters at P·V levels above 30 N·m/s. ZrO_2_ inclusions were more effective (WR = 6 × 10^−5^ mm^3^/Nm) than SiO_2_ ones since a TF formed with ZrO_2_ showed higher bearing capacity than in another case. The addition of soft nanoparticles, i.e., Bi_2_O_3_ and CuO, into the polymer matrix did not allow the formation of a strong and lubricious TF, according to Figure 15 *(PEEK; B-o-R; counterpart is the GCr15 steel; R_a_ = 0.27 μm; RT; P·V = 6–300 N·m/s).*

In [101] Ginzburg, B.M., et al. reported the influence of C60 fullerene (0.01–0.50 wt.%) on the tribological properties of PPS-based plastics with CFc (50 wt.%). The composite with 0.1 wt.% C60 exhibited the lowest linear WR level. The durability effect was related to healing of microcracks. Any cracks that reach the size of a fullerene molecule form covalent bonds with it as a result of the high electron-acceptor capability of C60 molecules. Further growth of forces that propagate the cracks breaks these bonds, causing the retraction of a C60 molecule into the microcrack. This process is accompanied by the formation of a great number of new covalent bonds of macroradicals with fullerene *(PPS; B-o-R; counterpart is a steel; R_a_ = n/d; RT; V = 1 m/s, CP = 5–60 MPa).*

### 3.8. Multicomponent Nanocomposites

Gao, C., et al. studied TF formation for ‘PEEK + 10 vol.% SCF + 8 vol.% Gr flakes’ composites additionally loaded with copper nanowires (0.5–5.0 vol.%) under WL conditions [102]. The sample filled with 0.5 vol.% nanowires exhibited the highest wear resistance. At P = 400 N and V = 0.2 m/s, CoF and WR levels were 0.13 and 4.5 × 10^−5^ mm^3^/Nm, respectively. The formed TF included PEEK, Gr, SCFs, and nanowires, as well as products of tribochemical reactions, i.e., both copper and iron compounds *(PEEK; B-o-R; counterpart is the 316 steel; R_a_ = 0.2–0.3 μm; RT; V = 0.2 m/s, P = 100–400 N).* In [103] Fan, S., et al. reported the influence of copper nanowires on functional properties of PEEK-based composites under WL conditions via the TF magnetic response. They showed that magnetic fields had a positive effect on the enhancement of their tribological performance, especially wear resistance. After loading with 1.0 vol.% nanowires, the composite’s WR enhanced by 65.07%. The TF formed with magnetic fields showed a lower elastic modulus and a superior easy-to-shear ability, illustrating that they endowed the TF with great lubricity and wear resistance *(PEEK; B-o-R; counterpart is the 316 steel; R_a_ = 0.2–0.3 μm; RT; V = 0.2 m/s; P = 100–400 N).*

Lin L. and Schlarb A.K. investigated the effect of various load conditions on the tribological performance of PEEK-based hybrid composites loaded with SCFs, Gr, and submicro and nanoparticles of TiO_2_, ZnS, SiO_2_ (5–10 wt.%) [104]. The lowest CoF value was achieved at P·V = 4 MPa·4 m/s, which was about 90% lower than that for neat PEEK. Accumulation of particles effectively reduced the direct contact between a counterpart and SCFs, similar to a contact of three bodies. During the sliding process, stresses transferred to fibers and impeded particles in front of them. As a result, debonding and removing SCFs took place *(PEEK, B-o-R; counterpart is the 100Cr6 steel; R_a_ = 0.2 μm; RT; V = 0.5–4.0 m/s; CP = 1–8 MPa).*

In [105] Zhang, G., et al. presented the friction and wear properties of neat PEEK and its nanocomposites in diesel and engine oil mediums. Nano-SiO_2_, SCFs, Gr, and PTFE were used as fillers. In both mixed and boundary lubrication regimes, the structure of the nanocomposites affected significantly their tribological performance. TF formation on the surface of a metallic counterpart played an important role in this phenomenon *(PEEK, B-o-R; counterpart is the 100Cr6 steel; R_a_ = 0.2–0.3 μm; RT; V = 0.2–2.0 m/s; CP = 5 MPa).* Zhang, D., et al. [106] studied roles of various fillers (SCFs 10 vol.%, Gr 8 vol.%, both WS_2_ and AlN nanoparticles 3 vol.% of each) on the tribological performance of PPS-based nanocomposites in low-sulfur diesel as a lubricant. The best tribological properties were determined for the ‘PPS + SCF + Gr + AlN’ sample with a specific WR level of 1.3 × 10^−7^ mm^3^/Nm and a CoF value of 0.08. Tribochemical reactions of PPS with a steel counterpart and AlN nanoparticles resulted in the formation of a robust TF *(PPS, B-o-R; counterpart is GCr15 steel; R_a_ = 0.2 μm; RT; V = 0.38 m/s; P = 100 N).*

Wang, Q., et al. investigated the effect of loading particles on the tribological properties of multicomponent PI-based composites with SCFs (10 vol.%), Gr (10 vol.%). and nano-Si_3_N_4_ (3 vol.%). [107]. With an increase in the P·V parameter, they enhanced (CoF = 0.75, WR = 4 × 10^−7^ mm^3^/Nm under DSF conditions at P = 500 N). The filled composites exhibited both better friction and wear behavior in an oil lubricator and showed worse tribological properties under WL conditions (as compared with DSF ones). In this case, an increase in the WR level was due to the fact that water might be first absorbed into voids at the interface between SCFs and the PI matrix, diffused slowly into the matrix that caused fall out of SCFs and their ‘cutting’ effect *(PI; B-o-R; counterpart is the GCr15 steel; R_a_ = 0.2–0.3 μm; RT; V = 0.431 and 0.862 m/s; P = 200–500 N).*

Panda, J.N., et al. studied the tribological potential of PAEK-based composites and their dry bearings [108]. The following fillers were loaded: SGFs (30 wt.%), Gr (10 wt.%) as a primary SL, and MoS_2_ and WS_2_ particles (10 wt.%) in both micro and nanoforms as secondary SLs. Filling with WS_2_ resulted in better performance than that for the MoS_2_ case. Nanoparticles were more efficient than microsized ones. The sample loaded with a combination of nano and microparticles of WS_2_ showed the best performance compared to other bearings, since a continuous thin TF reduced both CoF and WR parameters *(PAEK; B-o-R; counterpart is a mild steel; R_a_ = 0.2 μm; RT; V = 0.5 m/s; P = 100–500 N).*

## 4. Plane Contacts

### 4.1. Neat Polymers

In [109] Laux, et al. reported the influence of both contact pressure and molecular weight on TF formation and wear resistance of four PEEK-based composites in multidirectional tribological tests. As expected, rising pressure resulted in faster wear rates. The maximum TF thickness was observed on a wear track where the pin slept perpendicular to the counterpart roughness orientation and vice versa. The reason was mechanical abrasion and debris anchoring. The mean TF thicknesses were from 2.5 to 11.0 µm but did not show a direct correlation to WR levels because the lowest values were observed for the mean TF thicknesses less than 4 μm *(PEEK; P-o-D, Ra(PEEK) = 0.2 μm; counterpart is a steel pin of 56.6 HRC with a diameter of 20 mm; R_a_(steel) = 0.5 μm; RT; V = 0.1 m/s; P = 60 and 125 N; CP = 1.8 and 3.9 MPa; DSF).* Yan, et al. investigated a series of PEEK-based composites loaded with different PI contents (10–50%) [110]. Thermal endurance, friction-reducing capacity, and wear resistance were improved by filling with PI. WR values of the composites were dominated by tribological test temperatures rather than contact pressure levels. The wear mechanism changed from the abrasive type to the adhesive one at temperatures above T_glass_. The optimal PI content was 30% *(PEEK; P-o-D; counterpart is a stainless steel; R_a_ = 0.5 μm; RT, 100 and 200 °C; V = 0.5 m/s; P = 10 N; DSF).*

Wang, H., et al. evaluated the effect of porosity on the tribological properties of self-lubricating PEEK- and PPS-based composites [111,112]. Both CoF and WR parameters, as well as the contact temperature, decreased first and then increased with rising porosity. At its level of 21.1%, the lowest CoF, WR and contact temperature values were observed (with CoF level reduced by 85% and wear resistance improved by a factor of 1245). In this case, channels connected inside the composites that were capable of releasing grease uniformly and continuously onto the contact surface. Therefore, the self-lubricating effect of restored grease decreased both CoF and WR parameters *(PEEK; PPS; P-o-D; counterpart is the (0.42–0.50)% C-(0.17–0.37)% Si-(0.50–0.80)% Mn steel; R_a_ = 0.15–0.30 μm; RT; V = 1.4 m/s; P = 200 N).* For self-lubricating PPS-based composites, Wang, H., et al. showed that loading with 1 wt.% zeolite and impregnating with lithium-base grease in pores resulted in greatly lowering both CoF and WR parameters (0.024 and 1.79 × 10^−16^ m^3^/Nm, respectively). Compared to neat PPS, the CoF level reduced by 90%, and the WR value increased by 4.67 × 10^4^ times. Wear resistance enhanced by 3.6 times that for the ‘PPS + 30 wt.% NaCl’ composite. Due to both high load and temperature levels, grease squeezed from micro and nanopores provided the excellent lubrication effect [111] *(PPS; R-o-R; R_a_ = 0.15–0.30 μm; counterpart is the (0.42–0.50)% C-(0.17–0.37)% Si-(0.50–0.80)% Mn steel; RT; V = 1.4 m/s; P = 150 N; DSF.*

**Figure 16:**
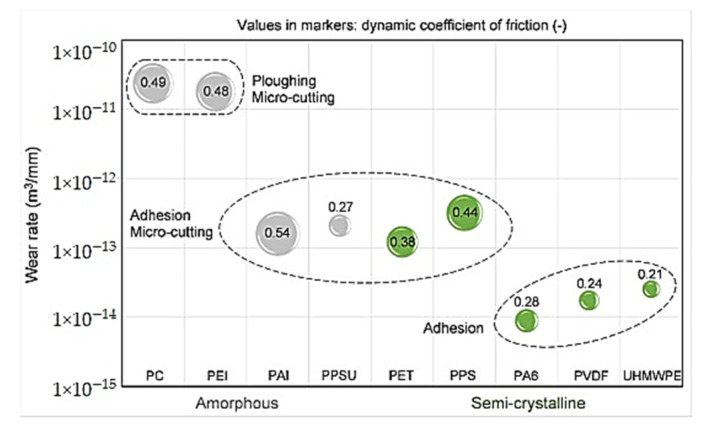
The WR vs. dynamic CoF diagram for amorphous and semicrystalline thermoplastics. Figure is reproduced with permission from reference [113].

In [113] Tóth et al. analyzed wear-induced crystallization of semi-crystalline polymers (PAI, PEI, PC, PPSU, PET, UHMWPE, PVDF, PPS, PA6). The wear mechanism of these thermoplastics was grouped into three different wear types such as abrasive, combined adhesive-abrasive, and adhesive (Figure 16). For the amorphous thermoplastics, both abrasive (PC, PEI) and adhesive-abrasive (PAI, PPSU) mechanisms were detected. The semicrystalline thermoplastics showed the adhesive-abrasive (PET, PPS) and adhesive (PA6, PVDF, UHMWPE) wear types. In the last case, the lowest CoF and WR parameters were observed for PA6, PVDF and UHMWPE. Three key parameters were directly related to the wear performance and the integrity of TFs: crystallinity, the relation of friction/bulk temperature to melting point, and the surface hardness *(PAI, PEI, PPSU, PPS; F-o-F; counterpart is the 100Cr6 steel; R_a_ = 0.2 μm; RT; V = 0.5 m/s, P = 10 kN; CP = 4 MPa; DSF).*

A series of papers [114,115,116,117,118] are devoted to the influence of various media on the tribological properties of HPP. In [115] Duan et al. observed the friction and wear properties of PPS, PES and PSU upon sliding: (1) without air cooling, (2) with air cooling, and (3) in water. Without air cooling, it was suggested that the CoF was mainly affected by the block polymer temperature controlled by the heat flow balance in the whole sliding contact system. Upon sliding in air, both PES and PSU WR levels raised continuously with increasing applied load. The minimum PPS WR level was recorded with the maximum CoF value. Under the water cooling and lubrication conditions, a liquid medium, consisting of water and debris, had an abrasive wear effect on the polymer. A higher PPS rigidity resulted in a comparatively coarser worn surface that caused a lower CoF level with a higher WR value compared with those for PES and PSU *(PPS; PES; PSU; P-o-D; counterpart is the 1Cr18Ni9Ti steel ring 15 mm wide with the outer and inner diameters of 25 and 20 mm, respectively; R_a_ = n/d; RT; V = 0.2 m/s; P = 25–250 N; CP = 0.75–7.50 MPa; no cooling, air cooling, and water conditions).*

Yamamoto et al. assessed the friction and wear performance of both PEEK and PPS in water [116]. In the PPS case, FeS formed on the mating steel surface as a result of its reaction with sulfur contained in PPS, which promoted the transfer or adhesion of the polymer to the counterpart. Consequently, PPS had a high ability for TF formation on the rubbing steel surface compared with PEEK. At the sliding surface, the PEEK hardness decreased under water lubrication, but this effect was not observed due to its immersion in water. The surface hardness reduction promoted greater WR levels in this case. *(PEEK, PPS; P-o-D; R_a_ = 0.5–0.8 μm; counterpart is a Cr-Mo steel pin with a diameter of 20 mm; RT; V = 0.012–4.000 m/s; CP = 0.25–2.00 MPa; WL).* In [117] Shen et al. reported the friction and wear performances of PAI on tungsten carbide (WC) in seawater for sealing applications. Under low loads, the ‘WC + PAI’ seal rings provided hydrodynamic lubrication at ultra-low CoF and WR levels. The lowest CoF value was reached at P = 90 N and V = 400 rpm. The P·V limit of 2.4 MPa m/s was shown at the end *(PAI; R-o-R; counterpart is a WC alloy; R_a_ = 0.5 μm; RT; V = 400 rpm, P = 45, 90, 180, 360 and 540 N, t = 8 h; DSF).*

Tailer et al. [118] analyzed the effect of counterpart materials on the sliding behavior of PI and PEEK in air, high vacuum, and hydrogen. In the last case, neat polymers possessed higher wear resistance for both studied counterparts from the 52100 and 304 steels, compared to air (especially PI as the most environmentally sensitive one). The 52100-steel counterpart promoted chemical reactions that enhance TF adhesion in hydrogen. An improved sliding behavior of neat PEEK took place on the 304-steel counterpart compared to another one, especially at high sliding speeds. The reason was a low thermal conductivity of austenitic steels, which contributed to softening in both PEEK and TF. Analysis of the counterpart surface yielded a conclusion that its influence was primarily associated with the chemical nature of the steel for PI and with the thermal conductivity of the counterpart for PEEK *(PEEK; P-o-D; counterparts are the 52100 and 304 steels; R_a_ =0.2 µm;RT; V = 0.2 and 1.0 m/s; P = 50 N; CP = 3 MPa; air, high vacuum and hydrogen with a purity of 99.999%).*

### 4.2. Reinforced HPP-Based Composites

Borruto A. [119] reported the possibility of using three types of PEEK-based composites in medicine, according to the formation of debris caused by tribological coupling with a cobalt-chrome alloy under both DSF and WL conditions. Demineralized water and human serum were applied as lubricants in the last case. The composite reinforced with 30 wt.% CFs showed a low WR level compared to that for neat PEEK since a little debris, including particles with average dimensions of about 0.1 μm, was produced in water and human serum lubrication, as well as under the DSF conditions. *(PEEK; P-o-D; counterpart is a CoCr alloy; R_a_ = 0.03–0.08, 0.08–0.30 µm; RT; V = 0.18 m/min; P = 20 N; CP = 0.12 GPa; DSF; demineralized water and human serum as lubricants).* In [120] Davim et al. studied the dependence of the tribological behavior of neat PEEK and the ‘PEEK + 30% CF’ composite on counterpart roughness, sliding speed, and contact stresses. It was found that the counterpart roughness was the most important factor affecting CoF values for both polymer and composite. This effect was about the same for neat PEEK (P = 48.6%) and the composite (P = 48.1%). The unidirectional sliding speed was more significant for neat PEEK (P = 34.0%) than for the reinforced polymer (P = 16.4%). Contact stresses had little to no effect on the frictional characteristics of both studied materials. The investigated dynamic conditions resulted in abrasive and adhesive wear mechanisms. The contact surface of the ‘PEEK + 30% CF’ composite pin had cavities around the fiber boundaries. Severe adhesive damages were observed due to the transfer of iron from a steel counterpart to the pin surface, as well as the polymer material backwards *(PEEK; P-o-D; counterpart is a steel; R_a_ = 0.5 µm; 37 °C; V and P have been set according to a 1 Hz sinusoidal function; WL).*

Davim et al. compared both CoF and WR parameters for neat PEEK, as well as the ‘PEEK + 30 wt.% CF’ and ‘PEEK + 30% GF’ composites [121]. The lowest CoF level of 0.21 was determined for the ‘PEEK + 30 wt.% CF’ sample. The ‘PEEK + 30% GF’ one showed its higher value of 0.36. Both studied composites possessed much better wear resistance than neat PEEK (2.4 and 6.4 vs. 23.9 mg, respectively) *(PEEK; P-o-D; counterpart is a carbon steel AISI 1045; R_a_ = 0.5 µm; RT; V = 0.25 and 0.75 m/s; P = 2.68 and 8.00 MPa; DSF).* Unal et al. reported the influence of both sliding speed and load on the tribological characteristics of the APK polymer and the ‘PPS + 30% GFR’ composite [122]. It was found that CoF values decreased linearly with increasing load for all investigated materials. Specific WR levels were on the order of 10^−5^ mm^3^/Nm for the APK polymer and the ‘PPS + 30% GFR’ composite, which had little dependence on applied loads and sliding speeds. Under the investigated conditions, sliding speeds exerted a stronger effect on the WR levels than loads for both materials *(PPS; P-o-D; counterpart is the AISI D2 steel; R_a_ = 0.11 µm; RT; V = 0.5–2.0 m/s; CP = 0.35–1.05 MPa; DSF).* In [123] Panda et al. evaluated the effect of the SGF amount (30 or 40 wt.%) in two composites, with synthetic Gr (10 wt.%) and PAEK (60 wt.% or 50 wt.%, respectively) on their thermal, mechanical, and tribological characteristics. Despite the high SGF contents, the tribological properties changed insignificantly. In general, both CoF and specific WR levels ranged between 0.04–0.08 and (3–8) × 10^−16^ m^3^/N × m, respectively. (They have decreased with raising the P·V parameters.) The limit P·V values were 112 and 100 MPa·m/s for the composites with 30 and 40 wt.% SGF, respectively. Loading with 30 wt.% SGF provided both lower CoF and WR levels at higher P·V limit values compared to the second investigated composite *(PAEK; P-o-D; counterpart is a mild steel disc; R_a_ = n/d; RT; V = 1.6–4.0 m/s, P = 700–900 N; DSF).*

A number of papers [124,125,126,127,128] are devoted to the influence of various media on the tribological properties of HPPs. In [124] Kumar et al. reported the effect of loading with AFs, CFs, GFs, and BFs on the tribological properties of PI and PTFE under DSF with dry or slurry erosion and high stress abrasive conditions. Lower CoF values do not always result in decreasing WR levels. For the studied materials, the main wear mechanisms were cutting and ploughing. Under both dry and slurry conditions, erosive neat polymers had the lowest WR value. Fracture and detachment of fibers were more pronounced under the dry and slurry erosive conditions. TF formation on a steel counterpart caused a decrease in the CoF values under the DSF conditions *(PI; PTFE; P-o-D; counterpart is C45 steel plate; R_a_ = n/d; RT; V = 0.1 m/s, P = 50 N; DSF, dry or slurry erosion and high stress abrasive conditions).* Tang, Q., et al. assessed the tribological characteristics of ‘CFR + PEEK’ composites when sliding on the Si_3_N_4_ counterpart in an aqueous medium [125]. The ‘CFR + PEEK’/Si_3_N_4_ friction pair showed both very low CoF (≤0.01) and WR levels over a wide range of sliding speeds and loads. (The first parameter has a greater effect than the second one.) For V < 3 m/s, the CoF values decreased as speed increased if the contact pressure was stable. For V > 3 m/s, the contact pressure increased with rising speed. The ‘CFR + PEEK’ TF on the Si_3_N_4_ counterpart and water lubrication could significantly reduce the CoF levels. In this case, the contact pressure affected the WR value of the composite through its influence on the surface temperature. The WR value enhanced mainly from material transfer caused by adhesion and mechanical plowing. TF formation alleviated friction. In addition, raising the P·V product resulted in increased surface temperatures and improved PEEK ductility *(PEEK; P-o-D; counterpart is Si_3_N_4_; R_a_ = 0.15 μm; RT; V = 1.36–3.70 m/s; CP = 0.45–0.92 MPa; DSF).*

In [126] Wang, Z., et al. investigated the ‘PAI + 30% CFR’ and ‘PEEK + 30% CFR’ composites under SWL conditions. Both samples were characterized by relatively low CoF and WR parameters at P = 100 N and V = 100 rpm. The ‘PEEK + 30% CFR’ one showed the best tribological characteristics. In the studied cases, CFs protected the materials from severe wear damage. The microcutting effect was observed on the worn surface of the ‘PAI + 30% CFR’ composite, while another one exhibited only mild scuffing *(PAI; PEEK; P-o-D; counterpart is a steel ring; R_a_ = 0.1 μm; RT; V = 0.5 and 1.0 m/s; P = 100 and 600 N; DSF; WL; SWL)* Harsha et al. analyzed the effect of different types of reinforcing fibers and fillers on the abrasive wear of PEEK, PEK, and PEKK upon multipass tribological tests on SiC abrasive papers of various grades [127]. Sliding distance, load, and grit sizes exerted a significant impact on the tribological characteristics. All studied polymers exhibited higher resistance to abrasive wear compared to their composites *(PEEK; PEK; PEKK; P-o-D; counterpart is the SiC abrasive papers; R_a_(#80) = 200 μm; R_a_(#120) = 175 μm); R_a_(#150) = 125 μm; R_a_(#220) = 50 μ; R_a_(#320) = 45 μm; R_a_(#400) = 40 μm; R_a_(#600) = 30 μm; R_a_(#800) = 20 μm; RT; V = 0.36 m/s; P = 2–10 N; DSF).* In [128] Yamamoto et al. evaluated both PEEK- and PPS-based composites reinforced with CFs and GFs in water. Under the WL conditions, the ‘PEEK + GF’ one had poor wear resistance. Loading PEEK with CFs significantly improved the tribological performance of the composite, which was improved for the PPS-based samples regardless of the type of loaded fibers. The greatest tribological properties were registered at high loads, under which neat PPS was worn out intensively. After loading both PEEK and PPS with 18 vol.% of the fillers, the fiber orientation affected wear resistance under mixed and hydrodynamic lubricating conditions, which was more efficient perpendicular to the sliding direction *(PEEK; PPS; P-o-D; R_a_ = 0.5 μm; counterpart is a Cr-Mo alloy pin; R_a_(steel) = 0.8 μm; 30±1 °C; V = 0.012–4.000 m/s; CP = 0.25–2.00 MPa; WL).*

### 4.3. Solid Lubricants

A number of research results have been published on the design of antifriction composites, in which low CoF levels and improved wear resistance have been achieved via loading with PTFE [129,130,131,132,133,134,135,136]. For example, Hufenbach et al. demonstrated that the ‘PEEK + 7.5 wt.% PTFE’ composite showed the best wear performance, while the ‘PEEK + 30.0 wt.% PTFE’ sample possessed the lowest CoF level [130]. The ‘PEEK + 7.5 wt.% PTFE’ composition was named the optimal one for the best combination of mechanical and tribological properties *(PEEK; P-o-D; counterpart is the 16MnCr5 steel pin with a diameter of 5 mm; R_a_ = 3.2 μm; RT; V = 0.35 m/s; P = 100–400 N; DSF).* In [132] Jones, M.R., et al. evaluated the role of the microstructure of PAI-based composites loaded with PTFE in amounts from 0 up 100 vol.%. It has been reported that the sample with 25 vol.% PAI was characterized by the highest WR  = 3 × 10^−9^ mm^3^/Nm over a distance of 360 km. A mechanical interlocking of both polymers occurred during the sintering process that contributed to the observed ultralow WR *(PAI; P-o-D; counterpart is a stainless steel; R_a_ = n/d; RT; V = 25.4–50.8 mm/s; P = 250 N; CP = 6.35 MPa; DSF).* Wang, H., et al. fabricated wear-resistant superhydrophobic PPS-based composite coatings [133]. The ‘PPS + PTFE’ surfaces had a nano/microstructure designed to possess the lowest surface energy clusters (inspired by the biomimic lotus leaves). The CF_2_-single bond group and PDMS showed a significant influence on the superhydrophobic coating formation. At 500 °C, weight losses of the ‘PPS + 45% PTFE’ coatings (both with and without PDMS) were less than 5% (related to improved heat resistance properties). Compared to other compositions of the studied coatings, the ‘PPS + 45% PTFE’ coating did not show any signs of failure for 15 h *(PPS; P-o-D; R_a_ = 0.15–0.30 μm; counterpart is a steel pin; RT; V = 0.47 m/s; CP = 1.4 MPa; DSF).*

In [134] Qi, H., et al. assessed the tribological properties of PI-based composites upon friction on counterparts from an aluminum alloy, a bronze bearing, and a steel bearing. When testing PI reinforced with aramid particles and PTFE on the bronze counterpart, a WR level was almost an order of magnitude lower than those for the other two studied cases. The composite exhibited much faster wear on the aluminum alloy counterpart under severe P·V products. Comprehensive analysis of tribochemical reactions showed that radicals originating from broken PTFE molecules chelated with bronze, forming a strong TF *(PI; P-o-D; counterparts are an aluminum alloy, a bronze bearing, and a steel bearing; R_a_ = 0.5 μm; RT; V = 1 and 2 m/s; CP = 4, 8 and 10 MPa; DSF).* Xie, G., et al. investigated ‘PEEK + PTFE’ composites, including those reinforced with potassium titanate whiskers (PTW) under various load conditions [135]. It was established that the reinforced specimens possessed much greater tribological properties. Both CoF and WR parameters decreased with increasing the filler content, especially when it exceeded 5 wt.%. The PEEK crystallinity was not the dominant factor influencing the tribological properties of the composites. At the initial friction stage, whiskers contributed to the abrasive wear type. Then, the formation of a thin, uniform, and tenacious TF on the counterpart, as well as embedding of worn-out free whiskers back into the composite surface were the key mechanisms of improving the composite wear resistance *(PEEK; P-o-D; counterpart is the AISI 1045 steel; R_a_ = 0.042 μm; RT; V =2 m/s; CP = 1.0, 1.5 and 2.0 MPa; DSF).* In [136] Zhu, Y., et al. showed the tribological behavior of porous ‘PPS + PTFE’ composites with both incorporated microporogen (NaCl) and mesoporous TiO_2_ whiskers. At P = 100 N, the lowest WR value was observed after loading with 7 wt.% MTiO_2_-W that was 6450 and 65 times lower than those for neat PPS and the ‘PPS + 10% PTFE’ composite filled with 30% microporogen, respectively. At P = 200 N, the lowest CoF and WR parameters were found for the sample loaded with 3 wt.% MTiO_2_-W (CoF = 0.019). Upon sliding, grease could be squeezed through nano/micro hierarchical pores under a coupling effect of load and friction heat. As a result, a stable layer was formed on the friction surface, providing the self-lubricating effect and improving wear resistance *(PPS; P-o-D; counterpart is a steel; R_a_ = 0.15–0.30 μm; RT; V = 1.4 m/s; P = 100 and 200 N; DSF).*

A number of studies [137,138,139,140] are devoted to the design of antifriction composites loaded with MoS_2_. As an example, Zalaznik et al. studied the effect of micro and nanosized MoS_2_ and WS_2_ particles in PEEK upon dry sliding on the 100Cr6 stainless steel [137]. It was shown that the addition of MoS_2_ and WS_2_ particles reduced CoF levels (down to 30%) regardless of their contents and sizes. However, nanosized particles required a higher content to form an effective TF with a low CoF value. TF formation was necessary to reduce WR levels (down to 51%) for all composites. In addition, loading with particles of all studied types caused enhanced hardness and improved wear resistance as a result. The XPS data showed that oxidation occurred upon the sliding process, reducing the beneficial effect of particles on the wear behavior *(PEEK; P-o-D; counterpart is the 100Cr6 steel; R_a_ = 0.5 μm; RT; V = 0.05 m/s; CP = 1 MPa; DSF).* Cho, M.H., et al. have investigated PPS filled with a molybdenum concentrate (MC), which is a mix of MoS_2_, SiO_2_, CuS, Al_2_O_3_, etc., as well as PTFE upon sliding on a steel counterpart [138]. In the presence of PTFE, a change in the MC content had the greatest impact on the WR reduction. CoF values varied in a narrow range from 0.27 up to 0.33. The lowest WR value was found for the ‘PPS + 17 vol.% MC + 10 vol.% PTFE’ composite at V = 1.5 m/s. For neat PPS, the WR level was 0.29 mm^3^/km. In the cases of the ‘PPS + MS + PTFE’ composites, TFs formed on the steel counterpart surface were very thin and uniform. Their XPS analysis showed that MoS_2_ decomposed into Mo and S to form both MoO_3_ and FeSO_4_ compounds. The presence of FeSO_4_ in the TFs indicates a tribochemical reaction between S and Fe on the counterpart, which contributed to the WR reduction between them. This retarded the TF loss from the counterpart and contributed to the WR reduction *(PPS; P-o-D; counterpart is a quench-hardened tool steel with a hardness of 55–60 HRC; R_a_ = 0.06, 0.10 and 0.14 μm; RT; V = 0.5, 1.0 and 1.5 m/s; CP = 0.65 MPa; DSF).*

In [139] Panda et al. assessed the tribological properties of composites based on PAEK (50 wt.%) with GFs (30 wt.%), natural Gr (10 wt.%), as well as loaded with SLs (10 wt.% of MoS_2_ and WS_2_ micro and nanoparticles). These characteristics enhanced as the sliding conditions got harder (until the limit P·V values of 100 MPa·m/s were reached). The composite filled with WS_2_ was characterized as the most wear resistant at moderate P·V products. Under the most severe conditions, the one with MoS_2_ showed the highest result. All studied composites proved to be excellent materials for tribological units (CoF = 0.04, WR = 2.2 × 10^−16^ MPa·m/s, and P·V = 84 MPa·m/s in safe modes). Loading with 3 wt.% MoS_2_ micro or WS_2_ nanoparticles greatly improved the tribological performance of the composites. In the WS_2_ case, this effect was less pronounced due to improper deagglomeration of these heavy particles. XPS studies found the partial formation of oxides including MoS_2_ or WS_2_ *(PAEK; P-o-D; counterpart is a mild steel disc; R_a_ = 0.1–0.2 μm; RT; V = 1.6–3.5 m/s; P = 700–900 N; CP = 22–28 MPa; DSF).* Song, J., et al. evaluated PI-based composites filled with aramid fibers, Gr, and MoS_2_ or PTFE [140]. Reciprocating sliding frequencies in a range from 3 to 11 Hz played a significant role in changing CoF levels of the composites, while increasing applied loads from 4 up to 6 MPa exerted little effect on this parameter. The ‘PI + Gr + AF’ composite possessed both lower CoF and WR values than those of the ‘PI + MoS_2_ + AF’ and ‘PTFE + AF + PI’ ones at 6 MPa and 7 Hz upon 8 h. TFs formed between a phosphor bronze pin and the ‘PI + MoS_2_ + AF’ or ‘PTFE + AF + PI’ composite surfaces changed both contact states and wear mechanisms that enabled a long service life of these tribological matings. *(PI; P-o-F; counterpart is a bronze; R_a_ = 0.1 μm; F = 3–11 Hz, CP = 4, 5 and 6 MPa; DSF)*

Graphite as a solid lubricating filler was used by the authors of [141,142,143,144,145]. For example, Padhan et al. investigated a combined effect of fillers (total 20 wt.%) in the ‘PAEK + SGF + Gr + PTFE’ composites, especially with increasing the PTFE content [143]. Loading with both PTFE and Gr greatly reduced WR levels down to 10^−16^ m^3^/Nm at low CoF values of about 0.03. In this case, thermal conductivity was the key factor for wear resistance of the composites. XPS analysis showed that chemical reactions occurred during tribological tests, which resulted in the formation of FeO, Fe_2_O_3_ and FeF_2_ on the worn surfaces. It was concluded that the choice of SL materials should be based on the film transferring capability, thermal conductivity, lubricity, and ability to degrade fiber-matrix bonding *(PAEK; P-o-D; counterpart is a mild steel disc; R_a_ = 0.1–0.2 μm; RT; V = 1.6–4.0 m/s; P = 700–900 N; DSF).*

In [144] Theiler et al. reported the influence of hydrogen-containing environments on the tribological properties of PI-based composites loaded with natural or synthetic Gr. The used PI were copolyimides based on benzophenonetetracarboxylic dianhydride (BTDA, PI1) and pyromellitic dianhydride (PMDA, PI2). The PI chemical structure had a major effect on the studied characteristics. While the wear process remained fairly stable for the ‘PI + 15 wt.% Gr (natural)’ composite under all investigated conditions, loading with the same amount of graphite showed a very low CoF level in hydrogen without any lubricants. The Gr lubricity was more effective in hydrogen than in moist air *(PI; P-o-D; counterpart is the 52100-steel disc; R_a_ = 0.08–0.22 μm; RT; V = 0.2 and 1.0 m/s; P = 50 N; DSF, vacuum of 10^−2^–10^−3^ Pa and a hydrogen environment of 10^3^, 10^4^ and 10^5^ Pa).* Rodriguez, V., et al. assessed the friction and wear response of neat PEEK, as well as the ‘PEEK + CF + Gr’ and ‘PEEK + CF + Gr + PTFE’ composites [145]. The solid lubricants effectively reduced CoF levels but not WR values. The lowest CoF of 0.28 was determined for the ‘PEEK + CF + Gr + PTFE’ sample due to the self-lubricating effect of PTFE. TF formation observed mainly for neat PEEK. Adhesion provided a protective effect against damages, giving better wear resistance. Abrasion was the main type identified for the composites filled with SCFs, PTFE, and Gr. This can be attributed to the PTFE soft nature which was poorly adhered to the counterpart surface and was rapidly removed as wear debris *(PEEK; P-o-D; counterpart is the 100Cr6 steel; R_a_ = 0.20 µm; RT; V = 20 and 50 mm/s; CP = 4.8 and 10.0 MPa; DSF).*

**Figure 17:**
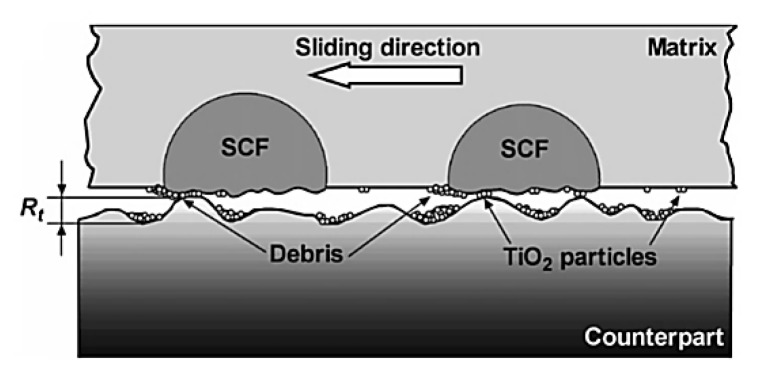
The rolling effect of submicrosized particles, protecting SCFs. Figure is reproduced with permission from reference [147].

In [146] Liu, L., et al. investigated ‘PEEK + SCF + PTFE + GE’ composites and analyzed the effect of loading with GE on their tribological behavior at various loads, sliding speeds, and temperatures. It was shown that the tribological characteristics were improved with increasing the GE content. The lowest CoF and WR levels were evaluated after loading with 2 wt.% GE. A thermal conductive network was formed in this case, contributing to wear suppression especially under severe tribological conditions. The mentioned multicomponent composite demonstrated both excellent lubrication performance and wear resistance. Moreover, the enhanced thermal conductivity enabled it to transfer friction heat from the contact, further contributing to improved wear resistance. *(PEEK; P-o-D; counterpart is the 304 steel; R_a_ = 0.15–0.30 μm; RT, 100 and 150 °C; V = 1.0, 1.5 and 2.0 m/s; CP = 1–4 MPa; DSF).* Jiang, Z., et al. reported a synergistic effect of both SCFs and TiO_2_ submicroparticles loaded in PPS for improving wear resistance [147]. The lowest WR value was obtained for the ‘PPS + 15 vol.% SCF + 5 vol.% TiO_2_’ composite but a better ‘PPS + 15 vol.% SCF + 6 vol.% TiO_2_’ composition was proposed by the authors. Under severe P·V products, an increase in sliding speed resulted in a relatively slower rise of the WR levels compared to the applied loads. This phenomenon indicated that a combination of appropriate P·V products could augment a hybrid reinforcing effect of these two fillers. As a result, a compact and smooth TF could be formed that reduces wear damages, according to Figure 17. *(PPS; P-o-D; counterpart is the 100Cr6 steel disc (LS 2542); R_a_ = 0.19 μm; RT; V = 1 and 3 m/s; CP = 1 and 3 MPa; DSF)*

**Figure 18:**
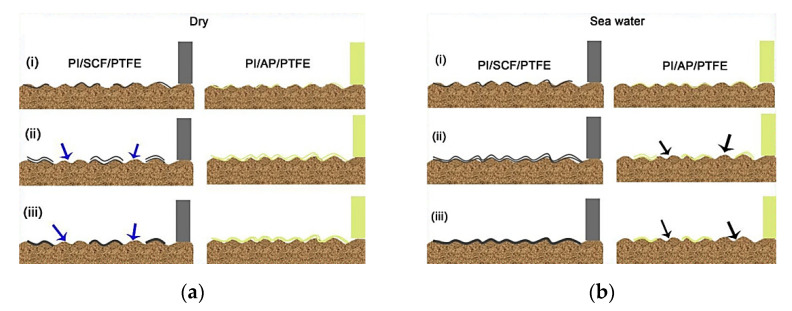
A schematic diagram of TF formation upon sliding of the ‘PI + SCF + PTFE’ and ‘PI + AP + PTFE’ composites on the copper counterpart surface under DSF (**a**) and SWL (**b**) conditions. The transfer process of PI composites to Cu surface consists of three steps: (i) initial transfer of wear debris, (ii) accumulation and removal of wear debris and (iii) compaction of wear debris. Figure is reproduced with permission from reference [149].

The tribological characteristics of SL-based composites have also been studied under various conditions and environments in [148,149,150]. As an example, Theiler et al. studied the sliding process for PEEK-based composites loaded with CFs, PTFE, as well as MoS_2_ or Gr in vacuum [148]. PEEK filled with MoS_2_ demonstrated better tribological properties compared to those for the one loaded with Gr only at low temperatures. At −80 °C, both negligible CoF and WR levels of the composite filled with MoS_2_ were recorded due to a thin polymer TF formed on a counterpart because of a higher MoS_2_ content on the composite surface. A transition from severe to moderate wear was observed in a P·V range from 0.01 up to 0.10 MPa·m/s. At RT in vacuum, the optimum performance was determined at P·V = 1 MPa·m/s. Depending on both temperature and sliding speed parameters, the composites filled with MoS_2_ showed great wear resistance and low CoF levels *(PEEK; P-o-D; counterpart is 304 steel discs; R_a_ = 0.2 μm; −80, −40, −20 and +20 °C; V = 0.01, 0.10 and 1.00 m/s; CP = 1 and 7 MPa; DSF).* In [149] Qi, H., et al. studied the influence of SCFs and aramid particles (APs) on the tribological performance of PI-based composites loaded with PTFE under both DSF and SWL conditions. APs were found more effective than SCFs for enhancing the tribological performance under DSF conditions. However, this statement was changed in the lubricant. Hard SCFs, exposed to the sliding interface, could cause debris removal and loss of the TF excellent lubrication ability (Figure 18). Under SWL conditions, weak interfacial interaction was beneficial to strong TF formation for the composites reinforced with SCFs rather than APs. Tribochemical reactions between PTFE and copper sustained the TF lubrication ability and prevented tribological corrosion under SWL conditions *(PI; P-o-D; counterpart is copper; R_a_ = 0.20–0.25 μm; RT; V = 0.5 and 1.0 m/s; CP = 1, 3 and 6 MPa; DSF, SWL).*

Unal, H., et al. showed the tribological properties for neat PAI, as well as the ‘PAI + 12% Gr + 3% PTFE’ composite, under two different conditions (with and without air cooling) [150]. With rising load and sliding speed, CoF and WR values increased slightly for neat PAI but decreased slowly for the ‘PAI + 12% Gr + 3% PTFE’ composite in both cases. For investigated P·V range, low CoF levels at high WR values were recorded under the air-cooling conditions. The average specific WR values of 8 × 10^−16^ and 4 × 10^−16^ m^3^N/m and CoF levels of 0.40 and 0.32 were determined for neat PAI and the ‘PAI + 12% Gr + 3% PTFE’ composite, respectively. The wear mechanism included both adhesive and abrasive processes *(PAI; P-o-D; counterpart is the 316L steel disc; R_a_ = 0.2 µm; RT; V = 0.5, 1.0, 2.0 and 3.0 m/s; P = 50, 100 and 150 N; DSF with and without air cooling)*.

### 4.4. Nanofillers

**Figure 19:**
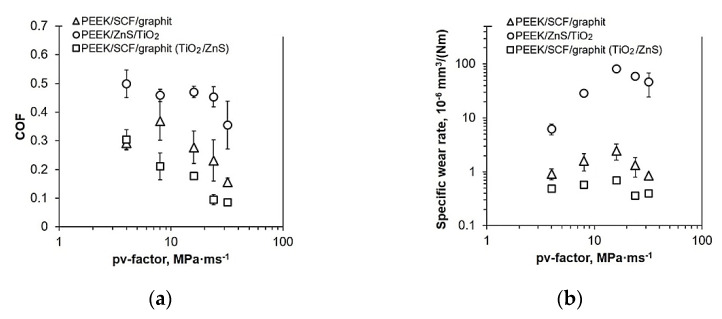
The mean CoF (**a**) and specific WR (**b**) levels of the PEEK-based composites under different P·V parameters at various sliding stages. Figure is reproduced with permission from reference [151].

In [151] Lin, L., et al. studied the role of nanoparticles on the tribological performance of PEEK-based composites. For example, these inclusions released from the ‘PEEK + ZnS + TiO_2_’ nanocomposite to a contact and improved the tribological properties under severe load conditions (at P·V > 8 MPa·m/s). In this case, released nanoparticles were sintered during the sliding process. As a result, a compact protective layer was formed on a steel counterpart and improved TF load-bearing capacity. Consequently, the direct contact between the composite and the counterpart were diminished and decreased both CoF and WR levels, according to Figure 19 *(PEEK; P-o-D; counterpart is the 100Cr6 bearing washer; R_a_ = 0.15 µm; RT; V = 0.1–4.0 mm/s; CP = 1–4 MPa; DSF).*

Hedayati, M., et al. demonstrated the effect of SiO_2_ nanoparticles on the tribological characteristics of both amorphous and semicrystalline PEEK-based coatings [152]. The semicrystalline neat PEEK sample possessed a higher hardness, as well as lower interfacial adhesion strength, CoF, and WR levels than those for the amorphous one. The addition of surface-modified SiO_2_ nanoparticles into PEEK resulted in raising the coating microhardness and interfacial adhesion. The WR levels were lower for both types of the nanocomposite coatings than those for neat PEEK, but their CoF values were slightly higher *(PEEK; P-o-D; counterpart is the 52100 steel; R_a_ = n/d; RT; V = 0.13 m/s; P = 3, 7 and 11 N; DSF).*

Cho, M.H. and Bahadur, S. devoted a number of papers to the influence of various types of nanoparticles on the tribological properties of PPS [153,154]. In [153] they studied thermal, dynamic mechanical, and tribological properties of ‘PPS + CNF’ composites. Loading with CNFs resulted in a slight change in the melting point, crystallization temperature, and T_glass_. WR levels of the reinforced composites sliding on a counterpart with *R_a_* of 0.13–0.15 μm were significantly lower than that for neat PPS. During tribological tests on a smoother counterpart (*R_a_* = 0.06–0.11 μm), the WR values were higher. Loading with CNFs did not affect CoF levels of the studied composites *(PPS; P-o-D; counterpart is a hardened tool steel of 55–60 HRC; R_a_ = 0.06–0.08, 0.09–0.11, and 0.13–0.15 μm; RT; V = 1 m/s; CP = 0.65 MPa; DSF).* The tribological behavior of PPS-based composites filled with CuO micro and nanoparticles under WL condition were reported in [154]. When sliding PPS on a steel counterpart in hydrodynamic, mixed, and boundary-lubricated regimes, CoF levels were 0.05, 0.05–0.20, and 0.25, respectively. After the addition of 20–30 vol.% CuO microparticles, the WR and CoF values decreased by about 90% and 75%, respectively, compared to those for neat PPS under the boundary-lubricated conditions. CuO microparticles as a filler were effective for improving wear resistance of PPS, but nanosized inclusions did not influence the result. In the boundary-lubricated sliding process, a TF on the counterpart formed but did not develop successfully for protecting the soft composite pin surface from harder metal asperities. The role of the TF was negligible in reducing wear damages *(PPS; P-o-D; counterpart is a hardened tool steel of 55–60 HRC; R_a_ = 0.09–0.11 μm; RT; V = 0.10 and 1.67 m/s; P = 9.8 and 58.8 N; WL).*

In [155] the tribological performance was assessed for ‘PPS + MWCNT’ composites as a function of loading with CNTs. The ‘PPS + 0.2% CNT’ sample possessed the lowest WR value of 15.86 × 10^−6^ mm^3^/Nm, while CoF levels almost did not change regardless of the CNT contents. The formation of homogeneous thin TFs contributed to a significant increase in wear resistance of the composites. In addition, a nonuniform MWCNT distribution was observed in the ‘PPS + 10% CNT’ sample, which played the key role in the tribological behavior of the composites *(PPS; P-o-D; counterpart is a hardened tool steel; R_a_ = 0.06–0.08, 0.09–0.11, and 0.13–0.15 μm; RT; V = 1 m/s; CP = 0.65 MPa; DSF).* Guo, L., et al. have investigated the effects of both CNTs and CNFs on the tribological performance of PPS-based composites [156]. No significant change in CoF values was found, depending on the type and content of the fillers. WR levels increased linearly with rising test distance, but no run-in stage was observed. Similar effects were reported for other counterparts finished to different roughnesses and textures. For the studied composites, the WR levels were lower than that for neat PPS in the initial friction period, but it was either the same or worse at longer sliding distances *(PPS; P-o-D; counterpart is a hardened tool steel of 55–60 HRC; R_a_ = 0.06–0.08, 0.09–0.11, and 0.13–0.15 μm; RT; V = 1 m/s; CP =0.65 MPa; DSF).*

**Figure 20:**
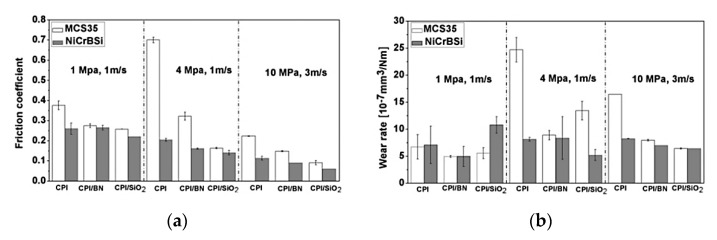
The mean CoF (**a**) and WR (**b**) levels of the PI-based composites upon sliding on the MCS35 and NiCrBSi counterparts. Figure is reproduced with permission from reference [157].

Qi, H., et al. analyzed the tribological behavior of various PI-based composites filled with both silica and hexagonal boron nitride (h-BN) nanoparticles upon sliding on the MCS35 steel and NiCrBSi alloy counterparts [157]. During tribological tests of the composites loaded with CFs and Gr on the NiCrBSi alloy surface, carbon-based TFs were formed that greatly reduced both CoF and WR levels (Figure 20). Nevertheless, no TF was formed at the contact with the steel counterpart. On the worn surfaces, chelation of polymer molecular radicals with metal analogs was revealed. Upon sliding of the hybrid composites filled with silica or h-BN nanoparticles, inclusions were released to the interface, significantly suppressing tribological oxidation of metal analogues. A tribologically sintered protective compact layer was formed as a mixture of residual polymer with oxidized debris *(PI; P-o-D; counterparts are the MCS35 steel and a NiCrBSi alloy; R_a_ = 0.25 µm; RT; V = 1 and 3 m/s; CP = 1, 4 and 10 MPa; DSF).*

In [158] Song, F., et al. presented results of the addition of nano-SiO_2_ and SLs (PTFE and WS_2_) in ‘PI + CF’ composites on their tribological performance over a wide range of load-speed parameters. Filling with nano-SiO_2_ particles enabled a significantly reduction in CoF levels regardless of the P·V products. Under mild P·V conditions, wear resistance of the ‘SCF + PI + SiO_2_ + SLs’ composite was improved through the synergistic effect of nanoparticles and the SLs. However, WR values enhanced significantly with rising P·V up to 59.26 MPa·m/s due to incorporating the SLs and their thermal instability. The fiber-matrix interface protection by nanoparticles, higher in both mechanical properties and thermal stability of SiO_2_, were responsible for enhancing the P·V limit up to 152.37 MPa m/s *(PI; P-o-D; counterpart is a stationary steel disc of 42–46 HRC; R_a_ = 0.2–0.3 μm; RT; V = 1–3 m/s; P = 100–900 N; CP = 5.65–50.85 MPa; DSF).* Ma, J., et al. loaded PI with mesoporous silica (MPS) and, consequently, reduced WR values by 90% compared to that for neat polymer due to the rapid formation of a high-quality TF because of some tribochemical reactions [159]. Results of XPS and ATR-IR analysis of the formed TF revealed the complexity of the tribochemical processes as well as their close connection with the TF formation mechanism. New polymer chain ends, formed inside the TF, enhanced its cohesion. The formation of carboxylate anion (-COO--), chelating to the steel surface in both bridge and bidentate forms, ensured building robust chemical bonds responsible for intact TF formation *(PI; P-o-F; counterpart is the GCr15 steel; R_a_ = 0.1 µm; RT; V = 0.5 m/s; P = 10 N; CP = 12.8 MPa; DSF).*

Golchin, A., et al. determined the tribological characteristics of ‘PPS + SCF + MWNT + Gr’ composites under WL conditions [160]. Loading with MWNTs and/or Gr marginally affected the friction and wear behavior of the composites. XPS analysis of the worn surfaces evidenced the formation of a reaction layer on the Inconel counterpart. EDS investigations revealed the presence of a FT with nanosized inclusions on the counterpart surface *(PPS; P-o-D; counterpart is an Inconel alloy; R_a_ = 0.3 µm; RT; V = 0.13 m/s; P = 80 N; CP = 5 MPa; WL).* In [161] Panda et al. studied the tribological properties of ‘PAEK + 30% SGF + Gr’ composites loaded with PTFE particles of micro and a combination of micro (7 wt.%) and nanosizes (3 wt.%). Compared to the ‘PAEK + 30% SGF + Gr’ composite, WR levels of the ‘PAEK + 30SGF + Gr + PTFE’ sample decreased by 18–30% while its CoF values was assessed between 2 and 18% due to the presence of PTFE particles (both nano and microsized). The composites showed a very high P·V level of 182 MPa·m/s with both low CoF and WR values (0.04 and 1.33 × 10^−16^ MPa·m/s, respectively). The formation of a polymeric TF on a metal counterpart surface was crucial in achieving the ultralow WR and CoF levels under DSF conditions *(PAEK; P-o-D; counterpart is a steel; R_a_ = 0.1–0.2 μm; RT; V = 1.6–7.0 m/s; P = 700–900 N; CP = 22–28 MPa; DSF).*

Iyer, S.B., et al. studied PAEK-poly (DMS) loaded with a low content of CNFs and various percentages of nano-HA particulates [162]. Filling with nano-HA particulates resulted in a significant reduction in WR values (down to ~10^−8^ mm_3_/N·m), compared to that for the composite with CNFs. This phenomenon was associated with the use of poly (DMS) that provided a cushioning effect in the matrix. Also, the phosphate grafting of poly (DMS) was implemented. In addition, the nanoscale of both fillers and their specific surface treatment using aminosilane for enhancing the matrix-filler interfacial bonding enhanced this effect. The maximum wear resistance was observed at the nano-HA contents of 7–8% *(PAEK; P-o-D; counterpart is the EN31 steel disc; R_a_ = 10 μm; RT; V = 400 rpm; P = 5, 30 and 60 N; DSF).* In [163] Chen, Y., et al. reported the influence of graphene functionalized by amines on the morphology and functional properties of PI-based nanocomposites. At 1.0 wt.% functionalized Gr in the polymer, CoF level decreased by 51.9% compared to that for neat PI (0.263 and 0.547, respectively). The minimum WR value of 1.895 × 10^−5^ mm^3^/N·m was typical for the composite with a filler content of 0.5% (by 46.3% greater than that for neat PI) *(PI; P-o-D; counterpart is a steel; R_a_ = n/d; RT; V = 0.072 m/s; P = 15 N; DSF).* Jia, Z., et al. evaluated the effect of nanoscale lamellar-structure expanded graphite (nano-EG) on the tribological properties of PI-based composites [164]. Under DSF conditions, this relationship was quite strong. Compared to neat PI, wear resistance of the sample loaded with nano-EG increased by a factor of 200 (1.8 × 10^−6^ mm^3^/N·m) at its content of 15 wt.%. The lowest CoF value was 0.135. A TF, formed on the surface of this self-lubricating composite, was smooth and continuous, which was the reason for the low WR level *(PI; R-o-D; counterpart is the 1045 steel; R_a_ = 0.1–0.2 μm; RT; V = 0.48 m/s; P = 200 N; DSF).*

In [165] Yijun, S., et al. demonstrated the friction and wear behavior of PI-based composites reinforced with CNTs and PTFE under DSF, water-, oil-, and alkali-lubricated conditions. Loading PI with CNTs and PTFE caused a great reduction in both CoF and WR levels (by 50 and 90%, respectively) compared to the neat polymer under the DSF conditions. On the contrary, the composites showed lower CoF and WR values in both oil and alkali lubricators. In the last case, the lowest WR value of 1.2 × 10^−6^ mm^3^/N·m was registered, which was 60% lower than under the DSF conditions. The worn surface of neat PI was characterized by strong adhesive wear, while the main wear mechanism was abrasive for the ‘PI + PTFE + CNT’ composite. The worn surfaces of the ‘CNT + PTFE + PI’ samples were smoother after sliding in oil or alkali lubricators than those under the DSF or WL conditions *(PI; P-o-D; counterpart is the AISI 1045 steel; R_a_ = 0.1–0.2 μm; RT; V = 1.4 m/s; P = 200 N; DSF, WL, oil- or alkali-lubrication).* Finally, Lin, G., et al. reported the influence of ZrO_2_ nanoparticles on the tribological properties of ‘PEEK + CF’ composites under both WL and SWL conditions [166]. Loading with two fillers remarkably improved WR levels of the composites in SWL, especially under high pressures. The high wear resistance of the ‘PEEK + CF + ZrO_2_’ sample was observed due to a synergy effect between nano-ZrO_2_ particles and CFs that carried a major part of loads during tribological tests and prevented severe wear damages of the matrix. Loading with ZrO_2_ nanoparticles greatly inhibited failure of CFs through reducing the stress concentration on the CF interfaces and shear stresses between two sliding surfaces. WR levels of the hybrid composites decreased with rising loads and sliding distances under the SWL conditions, while both low CoF and WR values were determined at high sliding speeds *(PEEK; P-o-D; counterpart is the 4Cr13 steel disk; R_a_ = 0.038 μm; RT; V = 2 m/s; CP = 1–8 MPa; WL).*

## 5. Discussion and Concluding Remarks

Based on the analysis of the cited papers within the framework of the review, as well as considering the authors’ own experience in conducting research on HPP-based composites [167,168,169,170], a number of conclusions have been drawn.

HPPs do not possess negligible CoF levels due to their high strength and cannot be classified as antifriction materials. For their use in friction units, the interface in the tribological mating is to be provided with a (liquid) lubricating medium. In this case, according to the classical patterns of tribology, rising wear resistance at a low CoF value is determined by the ability to retain a lubricating film on the contact surfaces. Materials that increase surface energy or containing functional groups contribute to this phenomenon.

Under DSF conditions, a harder counterpart, which contains asperities on its surface, affects the softer polymer surface, contributing to its wear. A part of debris is transferred and fixed on the counterpart or/and polymer (composite) surfaces, depending on the tribological loading conditions (roughness, P·V product, temperature, medium, interfacial adhesion, activity of fillers, and the counterpart, etc.). This is classified as a TF formation process in both cases. Changing tribological properties are determined by TF evolution, including its replenishment with newly formed debris from the initial polymer and the oxidized or fractured material. Filler particles can contribute to strengthening such a film, as well as its adherence on the sliding surfaces in the tribological contacts.

In a liquid medium (under the boundary lubrication), the tribological properties of fiber-reinforced HPP-based composites are greatly determined by the effect of this medium on the polymer matrix or the interface with reinforcing inclusions. In doing so, the wear intensity may be increased due to degradation of the composite structure on the sliding surface even at low CoF levels. Under severe tribological test conditions, the wear process can be transformed into a (micro)abrasive type due to rising temperature and local evaporation of the liquid medium.

The analysis of the above tribological test results on both HPPs and their composites using the point, line, and flat schemes has enabled us to state the following. The variety of the used fillers, the test conditions, and the achieved tribological properties show that the optimal compositions of the HPP-based composites for the given applications are highly case sensitive. There is no universal composition that has advanced functional characteristics under any circumstances. In the opinion of the authors, the following factors play an important role in the achieved tribological properties with the applied classification principle (see summary of the HPP composite studies in Table S1). The discussion assumes that a softer polymer composite slides on a harder counterpart.

### 5.1. Point Contacts

In the vast majority of cases, this scheme is implemented by sliding a smooth hard counterpart on a softer HPP-based composite according to the ‘ball-on-disk’ scheme. For neat HPPs, high CoF levels are typically observed, so even a smooth ball wears the polymer intensively. With a low roughness (Ra ~ 0.02 µm) of the hard counterpart surface, the TF purpose narrows to form smooth sliding contacts. The counterpart contact area is small, and both high specific pressure and local temperatures in the contact contribute to the TF adhering. The TF source is the entire sliding surface, so a large amount of debris is not required to form it. Especially with the addition of solid lubricant particles, this enables the achievement of extremely low wear rates at negligible CoF values. Under severe tribological test conditions (high P·V products), wear resistance is limited by the HPP matrix stability, the bonding strength with fillers, the ability of the TF to be adhered in the tribological contact zone, etc.

### 5.2. Line Contacts

Most studies of this contact type have been performed according to the ‘block-on-ring’ scheme. Compared to the previous case, the specific pressure is lower and the key differences are: (1) raising the number of asperities due to enhancing the contact area (which causes increasing CoF levels); (2) generally rougher counterpart surfaces (Ra > 0.1 µm); (3) due to its high roughness, a protective TF over the entire counterpart sliding surface needs to be formed from the wear debris; (4) the role of solid lubricant inclusions is significantly leveled, while an increase in wear resistance can be achieved by improving strength (reinforcing) of HPP-based composites. However, it is the possibility of TF formation and adherence on the harder counterpart, realized due to the chemical activity or increased surface energy of the filler, which enables the reduction in the CoF values. An alternative way is the formation of a thin modified (strengthened) layer on the wear track surface, which facilitates sliding and reduces wear intensity.

### 5.3. Flat (Area) Contacts

This kind of tribological contact is predominantly implemented with the ‘pin-on-disk’ scheme. However, a harder counterpart (disk) rotates relative to a fixed softer pin, unlike point contacts. The disc roughness is predominantly comparable to that for the ‘block-on-ring’ scheme (Ra > 0.1 µm). An increase in the contact area and the number of asperities on the sliding surfaces impose further restrictions on the structure and properties of polymers (composites). In this regard, the TF role also increases, which can be formed not only on the sliding surface of the hard counterpart (covering hard asperities), but also on the flat pin surface. In the first case, debris should form a continuous film that can adhere on the hard counterpart (at the running-in stage). Additionally, it is necessary to minimize the transfer of (metal) wear particles from the hard counterpart to the polymer pin to avoid enhancing CoF levels due to the solid-on-solid friction. On the other hand, the possibility of forming a modified reinforced layer on the sliding surface of the HPP pin is a way to increase both the CoF and WR values.

As a final conclusion, it should be noticed that a challenge of experimental methods for analyzing TFs, stated by K. Friedrich in [3], is effectively solved in recent studies by the XPS method, which enables to accurately determine their compositions. Although this can be done only after the completion of tribological tests, not only does it allow us to more accurately interpret results (considering specific conditions and loading schemes), but also to design HPP-based composites that form required TFs performing their preset functions.

## Supplementary Materials

The following are available online at https://www.mdpi.com/article/10.3390/polym14050975/s1, Table S1: Summary on tribological studies of HPP composites.

## Nomenclature

ABS	acrylonitrile butadiene-styrene
AF	aramid fibers
AGNS	amine-functionalized graphene nanosheets
AM	additive manufacturing
APK	aliphatic polyketone
APTES	aminopropyltriethoxysilane
BF	basalt fibers
B-o-D	Ball-on-Disk
B-o-F	Ball-on-Flat
B-o-P	Ball-on-Prizm
CFc	carbon fabric
CF	carbon fiber
CNF	carbon nanofibers
CNT	carbon nanotube
CoF	coefficient of Friction
CSL	calf serum lubrication
DSF	dry sliding friction
DMS	dimethylsiloxane
EBA	ethylene butyl acrylate
EDS	energy dispersive X-ray spectroscopy
EG	expanded graphite
FG	fluorinated graphene
FGO	fluorinated graphene oxide
F-o-F	Flat-on-Flat
GFs	glass fibers
GN	graphene
GNPs	graphene nanoplatelets
Gr	graphite
HA	hydroxyapatite
HAP	hertzian average pressure
HPP	high performance polymers
ILFG	ionic liquid functionalized graphene
LF	loading force
LRV	linear reciprocating velocity
LWF	long woven fibers
MPS	mesoporous silica
MHS	maximum Hertzian stress
MWCNTs	multi-walled carbon nanotubes
N/D	no data
P	applied load
PA6	polyamide 6
PAEK	polyaryletherketone
PAI	polyamide-imide
PAN	polyacrylonitrile
PBI	polybenzimidizole
PC	polycarbonate
PEEK	polyetheretherketone
PEI	polyetherimide
PES	polyethersulfone
PET	polyethylene terephthalate
PI	polyimide
P-o-D	Pin-on-Disk
P-o-F	Pin-on-Flat
PPL	polyphenylene
PPS	polyphenylene sulfide
PPSU	polyphenylsulfone
PSU	polysulfone
PTCDA	perylene-3,4,9,10-tetracarboxylic acid dianhydride
PTFE	polytetrafluoroethylene
PTW	potassium titanate whiskers
PVDF	polyvinylidene fluoride
RF	reciprocating friction
RH	relative humidity
R-o-D	Ring-on-Disk
R-o-R	Ring-on-Ring
RR	rotation radius
RS	reciprocating sliding
RT	room temperature
SGF	short glass fibers
SCF	short carbon fiber
SEM	scanning electron microscopy
SL	solid lubricant
SROF	short randomly oriented fibers
SWL	sea water lubrication
TF	transfer film
V	sliding speed
UHMWPE	ultra-high molecular weight polyethylene
WL	water lubrication
WR	wear rate
XPS	X-ray photoelectron spectroscopy
