# Supplementary material for: High Performance Polymer Composites: A Role of Transfer Films in Ensuring Tribological Properties—A Review
Source: Polymers (Basel). 2022 Feb 28;14(5):975. doi: 10.3390/polym14050975 (PMC8912496; doi:10.3390/polym14050975)
Supplement: Supplementary file 1 [file polymers-14-00975-s001.zip › polymers-1598633-supplementary.pdf]

polymers

MDPI

Table S1. Summary on tribological studies of HPP composites

HPP matrix	Counterpart	Contact scheme	Conditions	Reference
		**Point contact**		
PEEK	100Cr6 steel	B-o-D	DSF, RT	[24], [33], [46]
PEEK	Stainless steel	B-o-D	DSF, RT	[28]
PEEK	AISI 52100 steel	B-o-D	DSF, RT	[30]
PEEK	GCr15 steel	B-o-F	DSF, RT	[25]
PEEK	100Cr6 steel	B-o-F	DSF, RT	[31]
PEEK	100Cr6 steel	B-o-P	DSF, RT	[29], [47]
PEEK/PBI	AISI 52100 steel	B-o-D	DSF, HT	[32], [37]
PEEK	SUS 304 steel	B-o-D	WL, RT	[34]
PEEK	Alumina	B-o-D	WL, RT	[58]
PEEK	CoCrMo	B-o-D	CSL, RT	[59]
PPS	100Cr6 steel	B-o-D	DSF, RT	[26]
PI	GCr15 steel	B-o-D	DSF, RT	[36], [48], [49], [52]
PI	100Cr6 steel	B-o-D	DSF, RT	[45]
PI	GCr15 steel	B-o-P	DSF, RT	[51]
PI	GCr15 steel	B-o-D	DSF, HT	[35]
PI	GCr15 steel	B-o-P	DSF, HT	[42]
PI	GCr15 steel	B-o-F	RF, RT	[27], [50], [53]
PI	GCr15 steel	B-o-D	SWL, RT	[54], [55], [56]
PI	Si_3N_4	B-o-D	DSF, RT	[44]
		**Line contacts**		
PEEK, PEI, PES	30CrMnSiA steel	B-o-R	DSF, RT	[65]
PEEK	AISI 316 steel	B-o-R	DSF, RT	[67], [69], [96], [102], [103]
PEEK	100Cr6 steel	B-o-R	DSF, RT	[80], [81], [82], [84], [85], [86], [104], [105]
PEEK	GCr15 steel	B-o-R	DSF, RT	[100], [106], [107]
PEEK	X10CrNiMoTi1810	B-o-R	DSF, RT	[68]
PPS	AISI 1045 steel	B-o-R	DSF, RT	[64], [70], [76], [88], [101]
PPS	titanium alloy	B-o-R	DSF, RT	[66], [77]
PI	AISI 1045 steel	B-o-R	DSF, RT	[61], [62], [71]. [89], [90], [91]
PI	GCr15 steel	B-o-R	DSF, RT	[72], [73], [74], [75], [78], [83], [92], [93], [94], [95], [97], [98], [107]
PI	440C steel	B-o-R	DSF, RT	[99]
PAEK	Mild steel	B-o-R	DSF, RT	[108]
		**Plane contacts**		
PEEK	AISI 1045 steel	P-o-D	DSF, RT	[136]
PEEK	GCr15 steel	P-o-D	DSF, RT	[60], [109], [110], [121]
PEEK	100Cr6 steel	P-o-D	DSF, RT	[119], [137], [145], [151]
PEEK	AISI 52100 steel	P-o-D	DSF, RT	[152]
PEEK	AISI 304 steel	P-o-D	DSF, HT	[146], [148]
PEEK	Si_3N_4;	P-o-D	DSF, RT	[125]
PEEK	GCr15 steel	P-o-D	WL, RT	[120], [126]
PEEK, PPS	Cr-Mo steel pin	P-o-D	WL,RT	[116], [128]
PEEK	52100 and 304 steels	P-o-D	air, high vacuum and hydrogen	[118]
PEEK	4Cr13 steel disk	P-o-D	WL, RT	[166]
PPS	100Cr6 steel	P-o-D	DSF, RT	[111], [112], [113], [133], [153], [154], [155]
PPS, PEI, PPSU	100Cr6 steel	P-o-D	DSF, RT	[113]
PPS, PES, PSU	1Cr18Ni9Ti steel	P-o-D	DSF, RT	[115]
PPS	AISI D2 steel	P-o-D	DSF, RT	[122], [136], [138]

PPS	100Cr6 steel	P-o-D	DSF, RT	[147]
PAEK	Mild steel	P-o-D	DSF, RT	[123], [139], [143], [161], [162]
PAI	Stainless steel	P-o-D	DSF, RT	[132]
PI	C45 steel	P-o-D	DSF, RT	[124], [158]
PI	Aluminum alloy, bronze, bearing steel	P-o-D	DSF, RT	[134]
PI	Bronze	P-o-F	DSF, RT	[140]
PI	MCS35 steel and a NiCrBSi alloy	P-o-D	DSF, RT	[157]
PI	AISI 1045 steel	P-o-D	DSF, RT	[164]
PI	Copper	P-o-D	SWL, RT	[149]
PI	AISI 1045 steel	P-o-D	WL, RT	[165]
